# Core-Extended Naphthalene Diimide Dyads as Light-Up Probes with Targeted Cytotoxicity Toward Tumor Cells

**DOI:** 10.3390/biom15020311

**Published:** 2025-02-19

**Authors:** Valentina Pirota, Erica Salvati, Carla Risoldi, Francesco Manoli, Angela Rizzo, Pasquale Zizza, Annamaria Biroccio, Mauro Freccero, Ilse Manet, Filippo Doria

**Affiliations:** 1 Dipartimento di Chimica, Università di Pavia, V.le Taramelli 10, 27100 Pavia, Italy; valentina.pirota@unipv.it (V.P.); mauro.freccero@unipv.it (M.F.); 2 Institute of Molecular Biology and Pathology, Consiglio Nazionale delle Ricerche, Via degli Apuli 4, 00185 Roma, Italy; erica.salvati@cnr.it (E.S.); risoldi.1679059@studenti.uniroma1.it (C.R.); 3 Istituto per la Sintesi Organica e la Fotoreattività, Consiglio Nazionale delle Ricerche, Via Gobetti 101, 40129 Bologna, Italy; francesco.manoli@isof.cnr.it; 4 Translational Oncology Research Unit, IRCCS-Regina Elena National Cancer Institute, Via Elio Chianesi 53, 00144 Rome, Italy; angela.rizzo@ifo.it (A.R.); pasquale.zizza@ifo.it (P.Z.); annamaria.biroccio@ifo.it (A.B.)

**Keywords:** naphthalene diimide (NDI), G-quadruplex ligands, fluorescent probe, DNA damage response, telomeric DNA

## Abstract

Within the framework of rational drug design, this study introduces a novel approach to enhance the specificity of small molecules in targeting cancer cells. This approach starts from the use of dyads merging into a single entity, a naphthalene diimide (NDI) and core-extended NDI (ceNDI), both known as G-quadruplex (G4) ligands and fluorescent probes. The strategy aims to leverage the unique diagnostic strengths of the ceNDI moiety featuring red emission by improving its binding affinity and target selectivity through inclusion in dyads built with different linkers. The newly developed NDI-ceNDI dyads are promising probes, as they exhibit fluorescence turn-on upon DNA recognition and induced circular dichroism signals dependent on DNA conformation. Both dyads have an excellent affinity for hybrid G4, with two orders of magnitude higher binding constants than those for ds DNA. Their high cytotoxicity on cancer cell lines further demonstrates their potential as therapeutic agents, highlighting the role of the linker in target selectivity. Specifically, only the dyad with the rigid triazole linker exhibits selectively induced DNA damage in transformed cells, compared to normal cells primarily targeting telomeric regions. Our findings shed light on DIPAC’s potential as a promising theranostic agent, offering insights into future developments in precision medicine.

## 1. Introduction

Guanine(G)-rich nucleic acids (NAs) are present in important regions of the human genome, like the telomeres at the end of the chromosomes and the promoter regions of several oncogenes [1,2,3]. These sequences are highly prone to organize into non-canonical NA structures, known as G-quadruplexes (G4s), well recognized to actively participate in crucial biological processes essential for cell replication and survival [2,4]. Consequently, G4s have become a topic of interest as potential targets for developing new therapeutic approaches. In the cancer context, the human telomere, characterized by a single-stranded overhang at the 3’end consisting prevalently of repeats of the GGGTTA sequence, assumes critical significance. The telomere shortening during cell replication has been associated with cellular senescence, and a telomeric DNA maintenance mechanism is mediated by a reverse transcriptase enzyme, called telomerase. Notably, the telomerase machinery is upregulated in about 85% of rapidly replicating cancer cells, endowing them with “immortality”. The activity of this enzyme is intricately linked to the structural arrangement of these telomeric G-rich sequences into G4s, presenting an opportunity for their targeting as a novel anti-cancer therapeutic strategy [5,6,7]. A similar scenario exists for other G-rich sequences, especially in the promotor regions of genes, whose expression depends on the conformation adopted by the latter sequence, either hybridized in ds DNA with the complementary strand or folded into the G4 structure. Probing the G4 conformation of G-rich NAs, whether telomeric or not, in vivo remains challenging. Balasubramanian [8] and Lansdorp [9] independently reported the use of G4-specific antibodies that bind with nanomolar affinity, enabling the visualization of the G4 structures in cells through fluorescence-based imaging. Their data strongly support the existence and biological significance of G4s, highlighting their potential “druggability” in human cells [10]. Other authors have suggested the use of fluorescent ligands, whose fluorescence lifetime is highly dependent on the conformation of the bound DNA [11,12]. In parallel to the search for G4 probes, it has been reported that ligands that bind to G4s in solution, stabilizing their structures, also interfere with the biological processes involving G-rich sequences. Consequently, molecular systems targeting G-rich NAs folding into G4 have been widely explored for developing new therapeutic and biomedical approaches. In this frame, Neidle’s group [13,14] and our research unit have explored naphthalene diimides (NDIs) and core-extended NDIs (ceNDIs) as very versatile platforms for the design of new molecular systems able to perform a variety of functions targeting NAs. We have demonstrated that tri- and tetra-substituted monomeric NDIs, as well as dimeric NDIs, are potent and reversible G4 ligands [15,16,17,18,19,20] and, when suitably functionalized, can also act as alkylating or cleaving agents targeting guanine-rich nucleic acid sequences capable of folding into G4 structures [21,22]. In addition to their remarkable ability to bind NAs, the NDI and ceNDI platforms exhibit optoelectronic properties that can be effectively tuned by an appropriately chosen substitution pattern. Indeed, electron-rich substituents on the aromatic core give origin to absorption and emission in the visible spectroscopic window [23,24,25], thus making them appealing not only as G4 ligands but also as molecular tools for fluorescence imaging applications [26,27] and photodynamic therapy (PDT) [28,29]. In this context, we have demonstrated the potential of NDI dyads as potent agents exhibiting very low IC_50_ values for several tumoral cell lines, coupled with their intriguing behavior as *turn-on* fluorescent conformation-selective G4 probes [18,30]. By conjugating red and blue NDI dyes, we have engineered a non-fluorescent NDI dyad that becomes red emitting upon G4 binding thanks to the disruption of intramolecular aggregation between the two NDI units. The fluorescence lifetime, which is significantly different for G4/dyad and dsDNA/dyad complexes, was suggested as a key feature for the development of new rationally engineered G4 sensors [18,31]. Demonstrating intriguing biological activity and excellent probing properties, these dyads performed as multifunctional agents of interest for theranostic applications.

**Chart 1:**
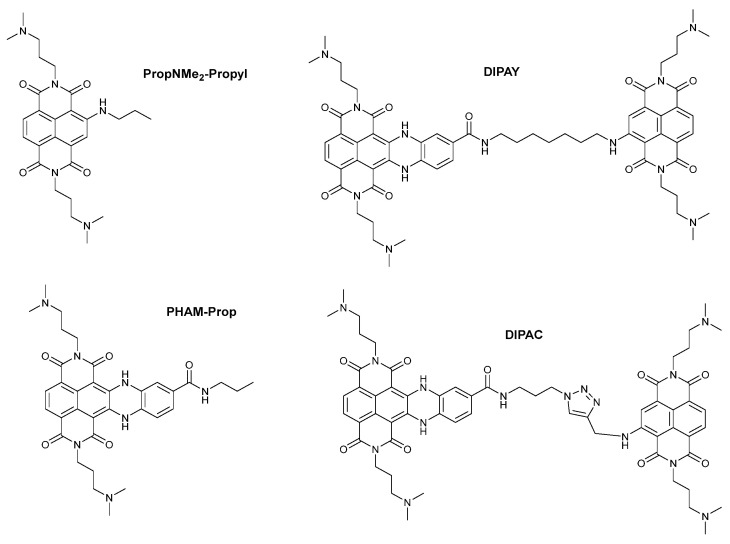
Chemical structures of the studied dyads (**DIPAY** and **DIPAC**), together with their monomeric models **(PropNMe2-Propyl** and **PHAM-prop**).

To improve the selectivity of these dyads towards cancerous cells, we present here a novel strategy for engineering NDIs as G4 ligands and probes, involving the combination of NDI and ceNDI into a single unit. The ceNDI moiety is endowed with red emission, expected to be quenched in the presence of NDIs with one amino substituent, due to intramolecular quenching processes, and should provide a unique diagnostic turn-on tool when recognizing the NA target. Two different spacers between the NDI and ceNDI units have been selected, one being conformationally flexible and the other rigid due to the triazole unit, to assess the impact of chemical structure on target selectivity (Chart 1).

The new NDI-ceNDI dyads, as well as their monomeric models, have been thoroughly analyzed. They display interesting fluorescence turn-on features upon DNA recognition, as well as an induced circular dichroism signal strongly depending on the DNA conformation. To assess their potential as anticancer agents, the synthesized ligands have been tested for their biological activity on selected cancer cell lines and models. The results obtained assert the potential of these dyads with a unique dual output as a DNA conformation probe and potential new therapeutic agent. A combination of therapeutic and diagnostic elements in a single small-molecular system displaying target selectivity offers a powerful strategy for precision medicine.

## 2. Materials and Methods

Solvents and reactants were purchased from Merck (Milan, Italy) and TCI (through Zentek srl, Milan, Italy) and were used as they were without further purification. HPLC analyses were performed using an Agilent system SERIES 1260 (Agilent Technologies, Santa Clara, CA, USA) with a SepaChrom ROBUSTA C18 column (3 µm, 4.6 × 50 mm, SepaChrom, Milan, Italy), using the following method: isocratic gradient over 2 min 95% of H_2_O + 0.1% TFA (5% CH_3_CN), gradually to 40% aqueous solvent over 6 min, then isocratic flow for 4 min (λ = 256 nm; flow 1.4 mL/min).

HPLC purifications were performed by an Agilent Technologies 1260 Infinity preparative HPLC provided with a diode array UV–vis detector. The column was a Waters XSelect^®^ CSH Prep C18 OBDTM (5 μm, 100 × 30 mm, Waters, Sesto San Giovanni, Italy), and the flow was 30 mL/min. Method A: isocratic gradient over 2 min 95% of H_2_O + 0.1% TFA (5% CH_3_CN), gradually to 60% aqueous solvent over 6 min, then to 0% aqueous solvent over 3 min (λ = 256 nm; 500 nm; 600 nm). Method B: isocratic gradient over 2 min 95% of H_2_O + 0.1% TFA (5% CH_3_CN), gradually to 60% aqueous solvent over 14 min, then to 40% aqueous solvent over 8 min (λ = 256 nm; 500 nm; 600 nm).

UHPLC-HRMS data were obtained using an X500B QTOF System (AB Sciex, Framingham, MA, USA) with an ESI ion source (UHPLC column: Kinetex 2.6 µm Biphenyl C18 column 50 × 4.6 mm, 100 Å, Phenomenex, Castel Maggiore, Italy). Isocratic UHPLC method using a 2:3 ratio of water to methanol, both acidified with 0.1% formic acid). ^1^H- and ^13^C-NMR spectra were recorded on a Bruker Avance 300 MHz (BRUKER, Billerica, MA, USA). Spectra were referenced to the residual solvent signal (D_2_O, 4.79 ppm).

### 2.1. Synthesis and Purification

**PHAM-Prop** [32] and **PropNMe_2_Propyl** [33] were synthesized as previously reported, validating the compliance of the structure by NMR characterization and the purity of the compounds by analytic HPLC. An HPLC purity > 97% was confirmed for all the final compounds tested; in addition, UHPLC-HRMS data were registered (see Supporting Information).

**PHAM-Prop** UHPLC-HRMS (positive mode). Found: 625.3075 *m*/*z*; calculated: 625.3085 *m*/*z*.

**PropNMe_2_Propyl** UHPLC-HRMS (positive mode). Found: 494.2749 *m*/*z*; calculated: 494.2762 *m*/*z*.

**Scheme 1:**
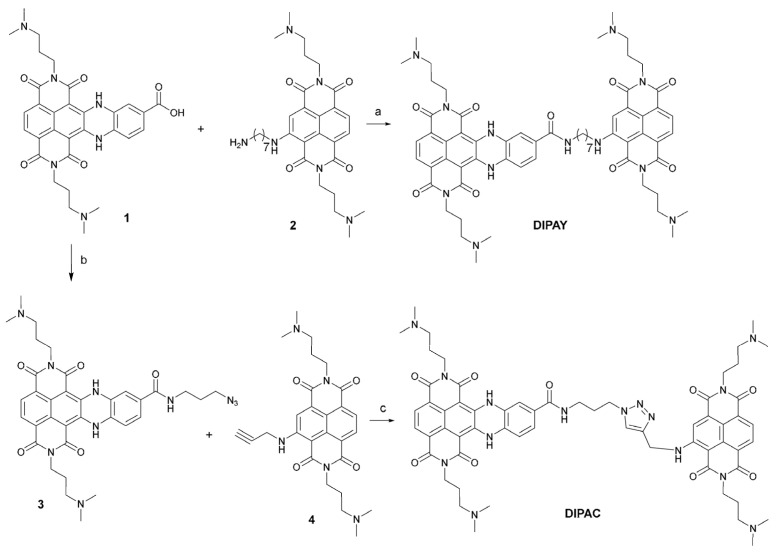
Synthesis of **DIPAC** and **DIPAY**: (a) 1 eq. of NDI **1**, 1.3 eq. of NDI **2**, 1 eq. of HATU, 1 eq. of HOBt, 4 equiv. of DIPEA, in DMF, rT, o.n.; (b) 3 eq. HATU, 12.5 eq. DIPEA, 3 eq 3-azido-1-propanamine hydrochloride in DMF, rT, o.n.; (c) 1 eq. of sodium ascorbate, 1 eq. of CuSO_4_ hexahydrate, 1:1 = tBuOH:H_2_O, rT, 3 h.

Synthesis of **DIPAY.** An amount of 162 mg of **1** (0.277 mmol), obtained as previously described [32], was dissolved in 6 mL of DMF, together with 1.3 equiv. of **2** (0.360 mmol, 203 mg), synthesized as previously presented (Scheme 1, step a) [30]. Subsequently, 1 equiv. of HATU (0.277 mmol, 105 mg), 1 equiv. of HOBt (0.277 mmol, 37.4 mg) and 4 equiv. of DIPEA (1.108 mmol, 193 µL) was added, and the mixture was left under stirring overnight at room temperature. The solvent was stripped under vacuum, and the final product, **DIPAY**, was obtained with a 45.3% yield after HPLC purification (Method A).

**DIPAY.** Analytic HPLC Rt = 5.85 min; 99.95%.

**DIPAY.** UHPLC-HRMS (positive mode): 1131.5760 *m*/*z*; calculated: 1131.5774 *m*/*z*.

^1^H-NMR (300 MHz, D_2_O): 8.13–8.00 (m, 3H), 7.93–7.91 (m, 1 H), 7.75 (s, 1H), 7.66–7.49 (m, 1H), 7.33–7.31 (m, 1H), 6.84 (s, 1H), 4.22–3.12 (m, 20H), 2.98 (s, 6H), 2.91 (s, 6H), 2.70 (s, 6H), 2.66 (s, 6H), 2.21–1.75(m, 18H).

^13^C-NMR (75 MHz, D_2_O): 164.9, 163.3, 163.2, 163.0, 162.9, 162.7, 162.6, 162.4, 162.1, 161.9, 161.2, 158.4, 155.4, 152.0, 139.1, 139.0, 138.9, 131.5, 130.9, 128.8, 126.6, 126.2, 125.3, 125.2, 124.8, 123.6, 123.5, 123.4, 123.1, 122.0, 122.0, 121.6, 121.1, 120.6, 118.2, 118.1, 115.9, 115.0, 114.2, 114.1, 110.6, 110.4,98.1, 95.8, 95.7, 95.5, 94.8, 55.3, 55.0, 54.9, 54.7, 42.8, 42.5, 42.5, 42.1, 39.7, 37.5, 37.3, 37.0, 36.8,36.4, 28.6, 28.2, 26.1, 25.5, 23.0, 22.8, 22.5, 22.4.

Synthesis of **DIPAC.** An amount of 40 mg of **1** (0.068 mmol), obtained as previously described [32], was diluted in 3 mL of DMF, adding 3 equiv. of HATU (0.204 mmol, 80 mg), 12.5 equiv. of DIPEA (0.340 mmol, 150 µL) and 3 equiv. of 3-azido-1-propanamine hydrochloride (0.204 mmol, 28 mg), obtained from commercially available 3-chloro-1-propanamine as previously described (Scheme 1, step b) [34]. The mixture was left stirring overnight at room temperature, and **3** was obtained after HPLC purification (Method B) with a 97.4% yield.

**3.** Analytic HPLC Rt = 6.60 min; 91.3% purity.

**3.** ^1^H-NMR (300 MHz, D_2_O): 7.68 (s, 2H), 7.21 (dd, J = 6.86 Hz, 1H), 6.95 (s, 1H), 6.70 (d, J = 8.35 Hz, 1H), 3.75–3.90 (m, 4H), 3.38 (t, J = 6.5 Hz, 2H), 3.30 (t, J = 6.6 Hz, 2H), 3.12 (t, J = 7.2 Hz, 4H), 2.83 (s, 12H), 1.99–1.91 (m, 4H), 1.84–1.76 (m, 2H) ppm.

^13^C-NMR (75 MHz, D_2_O): 168.9, 164.3, 164.2, 163.1, 163.0, 162.5, 158.8, 141.1, 140.7, 131.1, 129.1, 126.4, 125.1, 125.0, 124.8, 124.5, 121.2, 121.0, 118.1, 116.5, 115.2, 114.3, 96.0, 95.6, 55.1, 48.9, 42.7, 37.5, 37.0, 27.6, 22.6 ppm. 

An amount of 44.5 mg of purified **3** (0.066 mmol) was dissolved in 5 mL tert-butyl alcohol (tBuOH) in water in a 1:1 ratio, together with 1 equiv. of **4** (0.066 mmol, 32.3 mg), obtained as previously described [35]. Subsequently, 1 equiv. of sodium ascorbate (0.066 mmol, 13 mg) and 1 equiv. of CuSO_4_ hexahydrate (0.066 mmol, 10.5 mg) was added, and the mixture was left under stirring at room temperature for three hours (Scheme 1, step c). **DIPAC** was obtained after HPLC purification (method B) with a 95.3% yield.

**DIPAC.** Analytic HPLC Rt = 6.62 min; 100%.

**DIPAC** UHPLC-HRMS (positive mode): 1156.5466 *m*/*z*; calculated: 1156.5475 *m*/*z*.

^1^H-NMR (300 MHz, D_2_O): 8.12 (s, 1H), 8.02 (d, J = 7.8 Hz, 1H), 7.84–7.75 (m, 2H), 7.57 (d, J = 7.8, 1H), 7.23 (d, J = 8.3, 1H), 6.76 (d, J = 8.1, 1H), 6.53 (s, 1H), 4.12–3.90 (m, 4H), 3.89–3.84 (m, 2H), 3.75–3.65 (m, 2H), 3.64–3.56 (m, 2H), 3.33–3.23 (m, 4H), 3.14–3.02 (m, 4H), 2.93 (s, 12H), 2.79 (s, 6H), 2.76 (s, 6H), 2.47–2.32 (m, 2H), 2.15–1.86 (m, 8H) ppm.

^13^C-NMR (75 MHz, D_2_O): 166.6, 165.0, 164.0, 163.7, 163.3, 162.9, 162.8, 162.6, 162.4, 151.3, 142.4, 140.3, 139.8, 131.2, 130.7, 128.8, 128.6, 126.9, 125.9, 125.6, 125.5, 124.6, 124.1, 123.9, 123.8, 122.2, 121.4, 120.7, 119.6, 118.4, 116.6, 114.9, 99.2, 95.2, 95.1, 55.2, 55.1, 50.8, 42.8, 42.7, 42.7, 42.6, 39.5, 38.5, 37.5, 37.3, 37.1, 27.4, 22.8, 22.6, 22.5 ppm.

### 2.2. Sample Preparation for Solution Studies

For the spectroscopic measurements, we used pure water, as well as 10 mM K^+^ phosphate buffer of pH 7 with 100 mM K^+^. The concentration of the ligands was determined by weighing reliable amounts of the sample dissolved in known volumes of the buffer solution. For titrations, solutions with different NA concentrations were prepared by mixing aliquots of ligand and NA solutions in the buffer. The DNA, acquired by Eurogentec, used for this work is reported in the table below:
**NA****Sequence****Structure**ds265′-[CAATCGGATCGAATTCGATCCGATTG]-3′ds DNAhTel225′-A[GGGTTA]3GGG-3′hybrid G4hTel455′-GGG[TTAGGG]7-3′hybrid G4 dimerc-myc225′-[GAGGGTGGG]2GAAG-3′parallel G4kras5′-[AGGGCGGTGTGGGAAGAGGGA]-3′parallel G4

For the spectroscopic measurements, a 10 mM K^+^ phosphate buffer of pH 7.0 was used, with 100 mM KCl. Stock DNA solutions of a concentration of ca. 50 μM were prepared by dissolving the lyophilized compounds in a buffer solution containing 10 mM K-phosphate with 100 mM KCl of pH 7.0. The stock solutions were then heated at 90 °C for 15 min and slowly cooled to room temperature. The concentration of oligonucleotides was determined spectrophotometrically at 260 nm, using molar absorption coefficient values ε calculated based on the nearest neighbor model.

### 2.3. Absorption and Fluorescence Spectra

UV–visible absorption spectra were recorded on a standard commercial spectrophotometer, the Perkin Elmer λ950 (Perkin Elmer Italia S.p.a., Monza, Italy). CD spectra were recorded on the Jasco polarimeter J-715 (JASCO EUROPE S.r.l., Cremella, Italy), accumulating 4 spectra in the visible and 3 spectra in the UV with a scan rate of 50 or 100 nm/min and adjusting the cuvette path length to the optical density. Fluorescence spectra were measured on an Edinburgh Spectrofluorimeter FLSP920 (Edinburgh Instruments Ltd., Edinburgh, UK) using 1 nm steps and a 0.5–1 s dwell time. Slits were kept as narrow as possible to 2–4 nm in excitation and 3–6 nm in emission. Where necessary, a cut-off filter was used. Right-angle detection was used. All the measurements were carried out at 295 K in quartz cuvettes with a path length of 1 cm. All fluorescence spectra have been obtained for air-equilibrated solutions absorbing, in most cases, less than 0.1 at all wavelengths to avoid inner filter effects and the re-absorption of emission. Furthermore, they have been corrected for the wavelength-dependent response of the monochromator/PMT couple. The Φ_F_ value obtained for the compound **NDI-tri-propyl** [30] was used as a reference to determine the fluorescence quantum yield of the new compounds excited at 414 nm. Using the same solvents for all compounds and iso-absorbing solutions at the excitation wavelength, no corrections had to be made for absorbance or solvent refraction index, and we calculated the fluorescence quantum yields, Φ_F_, using the formula below, with A being the integrated area of the corrected fluorescence spectra:

Φ_F_ = Φ_F_^ref^ × A/A^ref^ (1)

### 2.4. Fluorescence Lifetimes

These were measured in air-equilibrated solutions with a time-correlated single photon counting system. A nanosecond LED source at 465 or 560 nm was used for excitation, and the emission was collected at a right angle at 620 nm using a long-pass cut-off filter at 590 nm. Decay profiles were fitted using a mono- or multiexponential function and deconvolution of the instrumental response.

I(t) = Σ_i_ a_i_ × exp(−t/τ_i_) and α_i_ = a_i_/Σ_i_ a_i_ (2)

<τ_av_> = Σ_i_ (a_i_ × τ_i_^2^)/Σ_i_ (a_i_ × τ_i_) (3)

### 2.5. Multiwavelength Global Analysis of Spectroscopic Titration Data

In the best complexation model, the binding constants, as well as single spectra of the complexes, were determined by means of a multivariate global analysis of multiwavelength fluorescence data, analyzing a set of spectra corresponding to different dyad/DNA mixtures. We used the commercial program ReactLabTM Equilibria (Version 1.1, Jplus Consulting Pty Ltd., East Fremantle, WA, Australia) developed in Matlab. The procedure is based on singular value decomposition (SVD) and nonlinear regression modeling by the Levenberg–Marquardt method. A matrix is created in Excel with all complete fluorescence spectra, and each spectrum is assigned to a specific DNA concentration. The file is launched from the ReactLabTM Equilibria application. Further parameters introduced before launching the optimization procedure are the ligand concentration, a binding model, approximate binding constants and the presence or not of fluorescence of the various species. The analysis proceeds to optimize the single binding constants as well as the spectra of the single species. It eventually affords upon convergence the individual fluorescence spectra of the complexes together with the final binding constants. The former were used to calculate the fluorescence quantum yields of the complexes. The software allows the comparison of experimental data and calculated values at single wavelengths to evaluate the goodness of the fit. Further statistical outputs include standard deviations for each fitted parameter, as well as sum-of-squares and standard deviations for the residuals.

### 2.6. Cell Culture

Human cervical cancer cells (HeLa; Lot No: 70030782), human osteosarcoma cells (U2OS; kind gift of Prof. Stefan Schoeftner) and human primary BJ foreskin fibroblasts (Lot No: 70063200) were purchased from the ATCC repository (through LGC Standards S.r.l., Sesto San Giovanni (MI), Italy). BJ HELT cells were obtained by transducing hTERT and SV40LT antigen genes with the subsequent retroviral vectors: pBABE-neo largeTcDNA (Addgene plasmid # 1780), and pBABE-hygro-hTERT (Addgene plasmid # 1773). All the cell lines were maintained in Dulbecco Modified Eagle Medium supplemented with 10% fetal calf serum, 2 mM L-glutamine and antibiotics at 37°C in 5% CO_2_. 

### 2.7. Cell Treatments and Viability/Survival Assays

Cells were seeded in 24-well plates at 5000 cells/well density. After 24 h, different concentrations of freshly diluted compounds were administered to the cells. Cell growth was monitored twice a day by an IncuCyte S3 Live-Cell System (Sartorius, Göttingen, Germany) and the analysis was performed with the Incucyte 2022B Rev3 Image Analysis Software (Essen Bioscience, Ann Arbor, MI, USA). For the assessment of survival, after 5 days of chronical treatment, cells were fixed in 2% formaldehyde, stained with crystal violet solution (0.5% solution in 20% methanol) and resuspended in isopropanol for absorbance measurement. The percentage of cell survival was determined as the percentage of OD_570_ in treated vs untreated samples. The IC_50_ was determined by Calcusyn 2.0 software on the survival curves. 

### 2.8. Immunofluorescence and FISH Analysis

Cells were fixed in 2% formaldehyde and permeabilized in 0.25% Triton X-100 in PBS for 10 min at room temperature at each endpoint and immunostained with Mouse Mab against phospho Ser 139 γH2AX (Merck Millipore clone JBW301) or Rabbit polyclonal anti 53BP1 (Novus 100-305) followed by anti-Rabbit Alexa fluor 555 and/or anti-Mouse Alexa fluor 488 secondary antibodies. Finally, coverslips were counterstained with DAPI and mounted on Mowiol. Immunofluorescence signals were acquired with a Leica DMIRE deconvolution microscope equipped with LAS V4.7 software.

## 3. Results and Discussion

### 3.1. Design and Synthesis

In this work, we have designed and synthesized two novel water-soluble NDI-ceNDI dyads (**DIPAY** and **DIPAC**, Chart 1) with unique optoelectronic properties. Together with their fluorescent properties, they have promising G4 binding features promoted by a synergistic interaction of both units with the target. To implement the potential of the recently published NDI dyads [18,30,31,33,36], we substituted an NDI moiety with a ceNDI unit, exploiting two distinct spacers for the conjugation of the two binding species. In the case of **DIPAY**, a highly flexible aliphatic chain was used, providing the dyad with elevated degrees of freedom. Conversely, **DIPAC** is distinguished by a more rigid spacer, which includes a triazole unit designed to bend and conformationally constrain the structure and serve as an additive binding portion. These conformational restrictions might reduce the likelihood of interacting with off-targets. **PHAM** and **PropNMe2-Propyl**, obtained as previously described [32,33], have been used as monomeric counterparts to properly compare the behavior of the new dimeric structures. Both the **DIPAY** and **DIPAC** synthetic protocols started from ceNDI 1, synthesized as previously described [32]. To obtain **DIPAY**, a coupling reaction between 1 and a slight excess of **2** [30] has been performed at room temperature with HATU as a condensing agent, in the presence of HOBt in basic DMF (Scheme 1, step a). To synthesize **DIPAC**, instead, we performed a first coupling reaction on ceNDI 1 with an excess of 3 equivalents of 3-azido-1-propanamine hydrochloride [34], in the presence of HATU as coupling reagent in basic DMF, to obtain ceNDI **3** (Scheme 1, step b). Copper-catalyzed Huisgen 1,3-dipolar cycloaddition between azide-modified ceNDI **3** and the alkyne-derivatives 4 [35], dissolved in 1:1 tBuOH:H_2_O mixture and in the presence of CuSO_4_·5H_2_O and sodium ascorbate was performed to afford the final dimeric compound **DIPAC** (Scheme 1, step c). 

### 3.2. Photophysical Characterization of the New Water-Soluble NDI-ceNDI Dyads 

A complete description of the photophysical properties of the water-soluble ceNDI monomers has been reported by Freccero et al. in [32]. ceNDIs have a strong tendency to aggregate, and solvent factors like pH, salt concentration and the presence of micelles affect their photophysical behavior. The fluorescence of ceNDIs in the aggregated state is completely quenched. In Figure S1, we compared the absorption spectra of the two new dyads (**DIPAY** and **DIPAC**) together with the spectra of two monomeric reference compounds, **PHAM-prop** and **NDI-tri-propyl** (Chart S1).

An absorption band with a vibronic signature in the 320–390 nm range is typical of the unsubstituted NDI aromatic core and assigned to π-π* transitions. The introduction of one amine substituent in NDI generates a second unstructured absorption band in the visible extending up to 580 mn of comparable intensity to that with a maximum at 370 nm, and this is due to a charge transfer transition. The ceNDI chromophore also has two absorption bands in the 390–450 nm and 500–640 regions, both with strong vibronic signatures due to transition from the ground state to the excited state with discrete vibrational energy [23,24]. The latter is due to higher rigidity in the core-extended chromophore that reduces the vibrational freedom of the molecule, giving rise to distinct vibronic levels in both absorption bands [23]. The higher-energy absorption band is likely due to aromatic core transitions, while the introduction of two N substituents in the 2,3 position of the ceNDI core gives rise to the lowest-energy visible absorption band and likely has a charge transfer origin, as already hypothesized for substituted NDIs [37]. Comparing the spectrum of the two ceNDI dyads with those of the monomers, we observe that the lowest energy vibronic peak is exclusively due to the ceNDI and not to the NDI unit of the dyad, affording a spectral region for exclusive excitation of the ceNDI unit. Moreover, the extension of the absorption to the red thanks to the ceNDI unit is crucial for multiple biomedical applications, as we can take advantage of the low-absorption window (650–950 nm) of tissues and blood. For **PHAM-Prop**, **DIPAY** and **DIPAC,** we have checked their aggregation behavior by performing dilution experiments monitoring their UV–visible absorption (Figure S2). In the case of **PHAM-Prop** and **DIPAY**, dilution clearly affects the shape of the absorption spectra, as well as the intensity, with an increase in the molar absorption coefficients for the most diluted 0.2 μM solutions (Figure S2). The relative intensity of the peaks at 595 nm and 550 nm drastically changes upon dilution, with the latter increasing in weight with aggregation. Differently, for **DIPAC** there seems to be no dilution effect. In the case of **PHAM-Prop**, the absorption spectra for dilution were analyzed by applying a simplified dimerization model, allowing a logK value of 6.6 to be calculated for the dimerization constant (see Figure S2 for spectra of the monomer and dimer). It was not possible to calculate a similar constant for **DIPAY,** as intermolecular aggregation adds to intramolecular interaction between the two chromophoric units, as already reported for the NDI-NDI dyads [18,30]. To check on intramolecular interaction, we further registered the absorption spectra of **PHAM-Prop**, **DIPAY** and **DIPAC** in 5 μM solutions with SDS micelles and compared them to those in pure water (Figure S1a and Table S1a). For **DIPAY and DIPAC**, with SDS the molar absorption coefficients increase and also the ratio A_595nm_/A_550nm_ of DIPAY, suggesting disruption of the intramolecular interactions (Table S1b). The different shapes of the absorption spectra of DIPAY and DIPAC without SDS are likely due to the more rigid and “kinked” triazole linker. In the case of the monomer **PHAM-Prop**, the increase in the ratio between the absorbances at 595 nm and 550 nm up to 1.56 indicates that SDS can promote complete disruption of the monomer aggregates, differently from the dilution test.

The fluorescence features of these molecules have been studied by means of steady-state and time-resolved techniques and further confirm intramolecular interaction in the dyads. Figure S2b shows the fluorescence spectra +/− SDS obtained for excitation at 565 nm, an isosbestic point of all the solutions examined. The two dyads have fluorescence quantum yields below 1% (Table 1) due to intramolecular quenching, in analogy with the homologous NDI dyads [18,30]. The addition of SDS gives a huge increase in the case of **DIPAY** and **DIPAC**. Interestingly, the fluorescence lifetime is different for excitation at 560 nm, where we mainly excite ceNDI, and 465 nm, where both chromophores absorb. In the absence of SDS, we observe a biexponential decay for excitation at 465 nm, with lifetimes that recall the values of the homologous NDI monomer (Table 1) [30]. For excitation at 560 nm, we observe a mono-exponential decay, with a lifetime of 3.2–3.3 ns assigned to the ceNDI unit. The addition of SDS gives only one lifetime value assigned to the ceNDI unit independently of the excitation wavelength, confirming that SDS affects the intramolecular interaction. Comparing excitation and absorption spectra (Figure S2c–e), we can conclude that energy transfer from NDIs to ceNDIs with the lowest excited state occurs and accounts for the single exponential decay. A second observation emerging from the excitation spectra of the compounds in water is that the maxima of the peaks at 594 and 547 nm are identical, so we can deduce the visible absorption maxima of the ceNDI unit in water to be at 594 and 547 nm.

### 3.3. Binding Study of the New Compounds to Model NAs 

**Figure 1:**
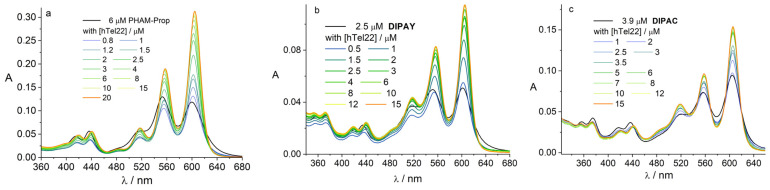
Absorption spectra of PHAM-Prop (**a**), DIPAY (**b**) and DIPAC (**c**) titrated with hTel22 in 10 mM K-phosphate buffer of pH 7 with 100 mM K^+^. Optical path 1 cm.

**Figure 2:**
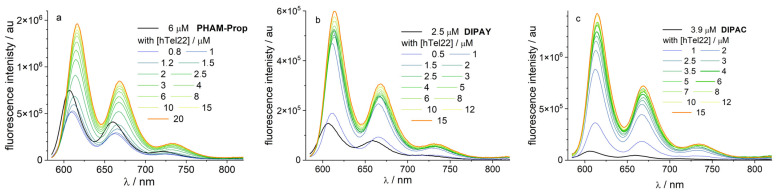
Fluorescence spectra of PHAM-Prop (**a**), DIPAY (**b**) and DIPAC (**c**) titrated with hTel22 in 10 mM K-phosphate buffer of pH 7 with 100 mM K^+^. Optical path 1 cm. Solutions were excited in isosbestic point at 574 nm (**a**), 572 nm (**b**) and 570 nm (**c**).

**Figure 3:**
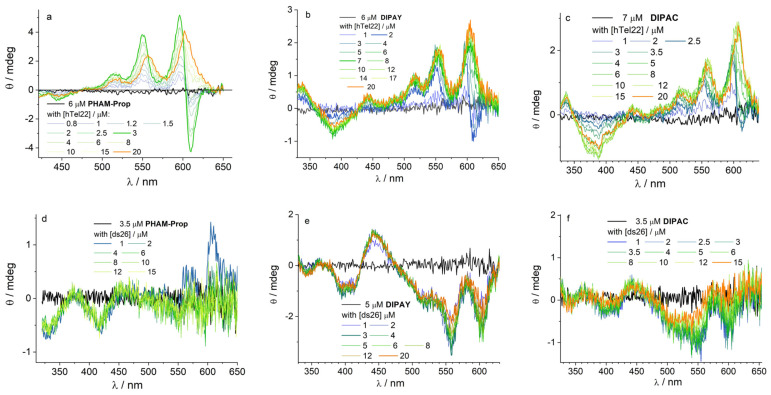
Visible circular dichroism spectra of **PHAM-Prop** (**a**,**d**)**, DIPAY** (**b**,**e**) and **DIPAC** (**c**,**f**) titrated with hTel22 (**a**–**c**) and ds26 (**d**–**f**) in 10 mM K-phosphate buffer of pH 7 with 100 mM K^+^. Optical path 2 cm.

The complexation behavior of **PHAM-Prop**, **DIPAY** and **DIPAC** towards four different conformations of DNA has been studied with different optical spectroscopic techniques. In particular, we examined the interaction with a self-complementary strand 5′-[CAATCGGATCGAATTCGATCCGATTG]-3′ as the ds DNA model, with the hybrid_1 G4 of the telomeric sequence hTel22 in the presence of 100 mM KCl, with the telomeric dimer hTel45 adopting a hybrid_1–hybrid_2 double G4 conformation and with the parallel G4 of c-myc22 present in the promotor of the MYC oncogene. The photophysical behavior of the complexes strongly depends on the type of DNA. Binding has been studied by titrating **PHAM-Prop**, **DIPAY** and **DIPAC** with different amounts of DNA, monitoring absorption, fluorescence and circular dichroism (CD) spectra, as well as the fluorescence lifetimes. Several pieces of information about their binding mode can be extracted from the photophysical features in the presence of DNA. The absorbance increases in the lowest energy peak and the change in the absorbance ratio of the peaks at ca. 600 and 550 nm are striking for the three compounds with telomeric DNA and less pronounced for parallel G4 and dsDNA except for **PHAM-Prop** (Figure 1 and Figure S3). These findings strongly suggest the disruption of the interaction between **PHAM-Prop** aggregated molecules and NDI and ceNDI units in **DIPAY** and **DIPAC** dyads upon DNA binding. The visible bands peaking at 558 and 607 nm exhibit a red shift of 11 and 13 nm, respectively, compared to the free ceNDI chromophore, suggesting the active participation of the ceNDI chromophore in DNA binding. The interaction of the dyad NDI chromophore with DNA appears less evident in absorption spectra due to the signal overlapping of ceNDI. Fluorescence intensity notably increases upon DNA addition in all cases, except for **PHAM-Prop** with ds DNA, albeit to varying degrees for the monomer and the dyads (Figure 2 and Figure S4). Remarkably, the fluorescence of the NDI chromophore in the dyad is totally quenched in the presence of DNA, and the long lifetime of the NDI for excitation at 465 nm disappears upon DNA addition, indicating NDI interaction with DNA. The maxima of the ceNDI fluorescence exhibit a red shift, and this holds also for the maxima in the excitation spectra. Therefore overall, absorption and fluorescence spectra confirm the interaction of both dyad chromophores with DNA. CD spectra in the UV region (Figure S5) show that the binding of **PHAM-Prop**, **DIPAY** and **DIPAC** to ds DNA induces a subtle change in the CD spectra, with the negative band at 250 nm becoming less negative with an increasing ligand-to-DNA ratio, suggesting a ligand-induced minor conformational change in the ds DNA. In the case of telomeric hybrid DNA, a decrease in the shoulder at 270 nm was observed, especially in the presence of excess ligand, indicating a possible shift to antiparallel G4 conformations. Ligand binding to parallel DNA does not affect DNA conformation. Interestingly, CD in the visible range gives information on the behavior of the ligands with the different NAs (Figure 3 and Figure S5). In the case of hybrid G4, an intense induced CD signal appears in the visible upon complexation of all three compounds (Figure 3a–c and Figure S5). In the presence of an excess of ligand, we can discern a bisignate signal at 600 nm, attributable to ceNDI, which disappears as the DNA concentration increases, with positive ellipticity persisting in the visible range. This confirms the existence of at least two complexes in solution, possibly with 1:1 and 1:2 (DNA/dyad) stoichiometry.

Excitonic coupling in a complex between two ceNDI units is responsible for the bisignate signal in the visible [38,39]. It does not necessarily require π-π stacking of the two chromophores, spectroscopically evidenced by band broadening in the absorption spectrum or quenching of fluorescence intensity, effects not observed for the dyad studied when interacting with the hybrid G4 structure [40]. The excitonic CD band is not observed for hTel45, a larger biomolecule where the chromophores likely remain distant. No visible CD signal appears upon complexation to parallel G4 DNA. This absence indicates that no electronic communication occurs between the two chromophores, or they have no restricted conformational freedom. A CD spectrum with an opposite sign is observed upon complexation to ds DNA (Figure 3d–f) [41]. Two dyads can be arranged in the hybridized 26 mer, adopting a helix with two complete turns. The alternation of the ellipticity sign from negative above 480 nm to positive in 420–480 nm may be indicative of the intercalation of the ceNDI chromophore, as it is known that mutually orthogonal transition moments (long and short axis vs long axis of the DNA base pair) of an intercalated chromophore have opposite induced CD signals. Intercalation is also in agreement with the reduced CD signal in ds DNA for the negative band at 250 nm and the fluorescence lifetime features [42]. As not all systems are endowed with useful CD signals in the visible, we eventually used the fluorescence data to determine the best complexation model and the binding constants of the most stable complexes (Table 2), as well as the individual spectra of the associated species (Figure S6). In the case of **PHAM-Prop**, we successfully included dimerization equilibrium during analysis with a logK value of 6,6. Complexation to DNA disturbs the dimerization equilibrium of **PHAM-Prop,** with annexed changing optical properties. The model compound **PHAM-Prop** has binding constants for the site of the highest affinity that is similar for G4 DNA and ds DNA, considering a binding constant with logK 7.6 for each site in the latter complex; so, the ligand **PHAM-Prop** is not discriminating any DNA conformation. Looking at the dyads **DIPAY** and **DIPAC**, they display a significantly lower affinity for ds DNA compared to the hybrid G4 DNA, looking at the 1:1 complex. This result valorizes our hypothesis that a ligand dyad structure can positively influence NA conformation selectivity compared to a single-unit ligand. The binding constant of **DIPAY** for hTel22 is among the highest values reported so far for NDI compounds binding to hybrid G4 DNA. This high value is also confirmed by the visible CD data analysis. Differently, the more rigid dyad **DIPAC** is not discriminating between G4 conformations, confirming the importance of the chemical nature of the linker in DNA binding.

We collected the fluorescence decays of **PHAM-Prop**, **DIPAY** and **DIPAC** in the presence of increasing DNA concentrations, directly exciting the ceNDI chromophore at 560 nm and performing a global analysis of all decays using a multi-exponential decay function. At this wavelength, only ceNDI fluorescence is observed. When excited at 465 nm, we also observe NDI chromophore decay, which disappears even at very low DNA concentrations, leaving only the ceNDI emission. The global analysis yields both the lifetime values (τ_i_) and the pre-exponential factors, a_i,_ that allow the calculating of the intensity-weighted average fluorescence lifetime, <τ_av_>, of each solution with the relative amplitude α_j_. In case of multiple lifetimes of the same fluorophore experiencing different environments like different binding sites, the α_j_ values in Equation (2) represent the fractional concentration of each emitting species. A two- or three-exponential decay function was required to achieve convergence for the global analysis of all fluorescence decays. The α_j_ value of the long lifetime, typical of the free molecule, strongly loses weight, and a species with a shorter lifetime appears in all cases, with the α_j_ value depending on the compound as well as the DNA (Figure S6 and Table S2). Overall, the trend in α_j_ values mimics the trend in species concentration, confirming the model emerging from the binding analysis. A global analysis is further completed, with the data in Table 3 showing the decay parameters for the solutions with excess DNA without free ligands. In all cases, a bi-exponential fit affords good results: the long lifetime of complexed **PHAM-Prop** is similar to the lifetime of the free molecule. Differently, the long lifetime is shorter for the dyads and depends on the DNA conformation. The existence of two lifetimes in the presence of DNA excess is indicative of at least two complexation sites that can be assigned either to the simultaneous presence of complexes with a different stoichiometry or to a situation with only one complex having binding sites with a similar affinity but different local environments (**PHAM-Prop** with hTel22, htel45 and ds26). Noticeably, the intensity-weighted average lifetime, <τ_av_>, is shorter for ds DNA compared to G4 DNA (Table S2), as well as the value of the short lifetime in ds26 complexes being identical to the lower limit of the TCSPC technique for the dyads (Table 3). These features are in line with the intercalation of the ceNDI dye suggested above. The fluorescence lifetime features fully confirm the complex binding behavior of the ligands, resulting in the co-existence of multiple complexes and/or multiple sites in single stoichiometry complexes.

### 3.4. Biological Studies

**Figure 4:**
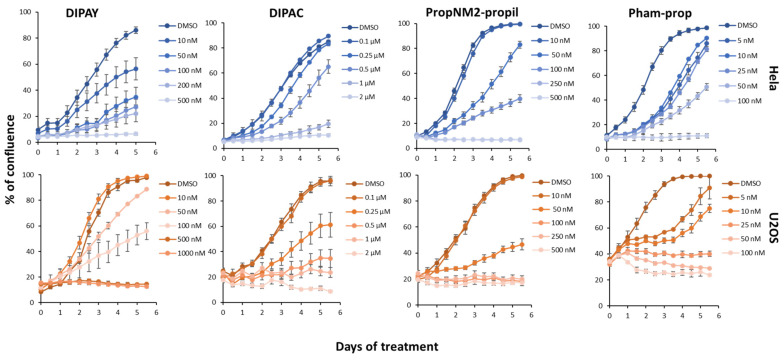
Hela and U2OS cells were seeded and chronically exposed to the indicated concentrations of the compounds. Growth was monitored by Incucyte every 12 h for 5 days. The curves report the % of confluence measured by live-cell imaging. One representative of three independent experiments with similar results.

**Figure 5:**
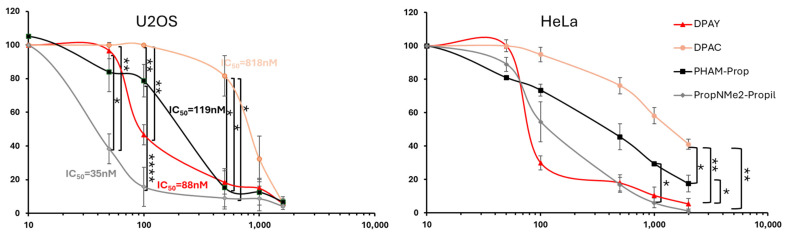
Hela and U2OS cells were seeded and chronically exposed to the indicated concentrations of the compounds. After 5 days, the cells were fixed and stained with crystal violet. The survival of the cells was calculated as the ratio of the absorbance measurements (OD_590_) in treated vs vehicle-treated samples. The mean of three independent experiments is shown. Bars are SD. Statistically significant differences were calculated with the two-way ANOVA test. * = *p* < 0.05; ** = *p* < 0.001; **** = *p* < 0.0001.

Based on previous results showing the significant cytotoxicity of NDI dyads in living cells and knowing the biological relevance of G4 stabilizing agents in cancer biology, we evaluated the biological activity of both monomers, **PropNMe_2_-Propyl** and **PHAM-Prop**, as well as both the **DIPAY** and **DIPAC** dyads in two different human cancer cell lines possessing long (U2OS) and short (Hela) telomeres. First, we chronically exposed cells to different concentrations of the compounds, and then we followed their growth in a real-time quantitative live-cell imaging system, quantifying the confluence of cell cultures for different treatments every 12 h (Figure 4). Notably, all the compounds induced cytotoxic effects on both cell lines at different dose ranges. Subsequently, based on the dose-dependent cytotoxicity of the compounds, we selected a sub-range of concentrations of each compound to determine the percentage of survival inhibition after treatment and the IC_50_ values, to compare their cytotoxicity in the two cell lines. As shown in Figure 5, the compounds showed very different IC_50_ values, and, more interestingly, compared to Hela, U2OS cells appeared more sensitive to all compounds except the **DIPAY**. Indeed, between the two dyads, the **DIPAY** resulted in being much more effective in terms of survival inhibition.

**Figure 6:**
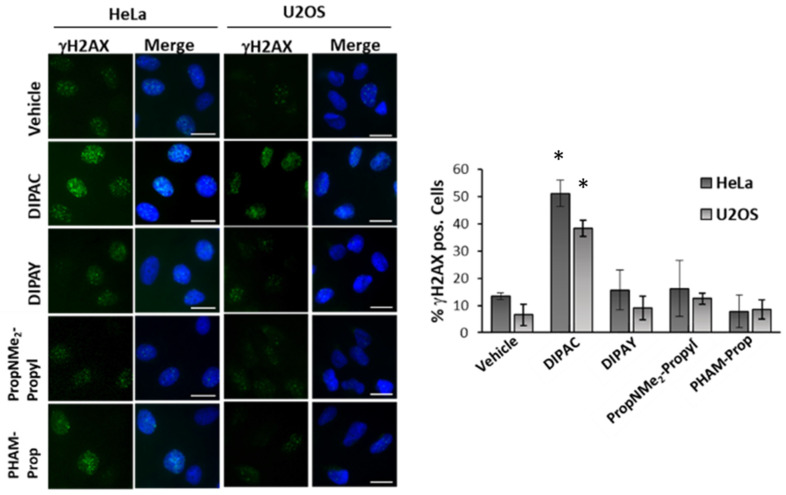
Hela and U2OS cells were seeded and treated with IC_50_ concentrations of the compounds. After 24 h, the cells were fixed and processed for immunofluorescence with an anti-γH2AX-specific antibody followed by a FITC-conjugated secondary antibody and counterstained with DAPI (representative images at 63× magnification are shown in the upper panels). The percentage of γH2AX foci-positive cells was scored and is reported in the histograms (lower panel). The graph reports the mean of three independent experiments. Bars are SD. Statistically significant differences were calculated by Student’s *t*-test. Scalebar = 10 μm. * = *p* < 0.05.

To ascertain if the cytotoxic effect of the compounds was dependent on DNA damage induction (DDR), we treated both Hela and U2OS for 24 h with IC_50_ doses of the compounds, and then we processed the cells for immunofluorescence with an anti-γH2AX-specific antibody (a well-established marker for DDR). The **DIPAC** dyad induced DDR activation in a percentage of cells that was consistent with its cytotoxic effect; instead, very surprisingly, despite their high cytotoxic effect, **DIPAY** and the monomers were not able to trigger DDR activation in either Hela or U2OS cells (Figure 6). The full DDR induction by **DIPAC** was also confirmed by the recruitment of 53BP1 at the sites of γH2AX phosphorylation (Figure S7).

**Figure 7:**
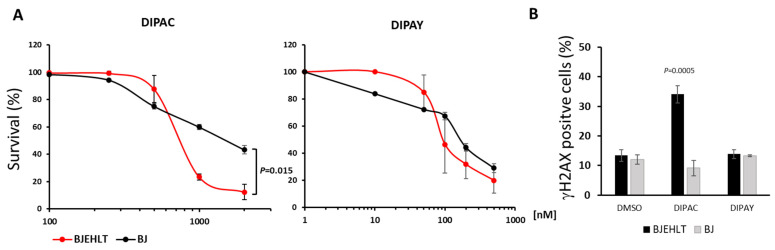
(**A**): BJ and BJEHLT cells were seeded and chronically exposed to the indicated concentrations of the compounds. After 5 days, the cells were fixed and stained with crystal violet. The survival of the cells was calculated as the ratio of the absorbance measurements (OD_590_) in treated vs vehicle-treated samples. (**B**): Cells exposed to an IC_50_ dose of the indicated compounds for 24 h were stained with an anti-γH2AX-specific antibody and counterstained with DAPI. The percentage of positive cells was scored and is reported in the graph. The mean of three independent experiments is shown. Bars are SD. Statistically significant differences were calculated by Student’s *t*-test.

**Figure 8:**
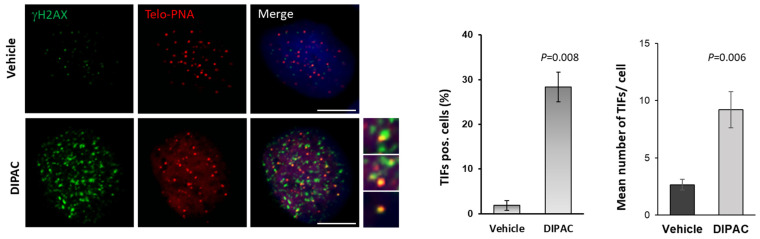
U2OS cells were seeded and treated with an IC_50_ concentration of the compound. After 24 h, the cells were fixed and processed for IF-FISH with an anti-γH2AX-specific antibody, followed by an FITC-conjugated secondary antibody, then hybridized with a Cy3-conjugated telo-PNA probe and finally counterstained with DAPI (representative images at 63× magnification are shown in the left panel). The percentage of TIF-positive cells (calculated as the percentage of nuclei displaying more than 4 co-localizations) and the mean number of TIFs per nucleus were scored and are reported in the histograms (right panel). The graphs report the mean of three independent experiments. Bars are SD. Statistically significant differences were calculated by Student’s *t*-test. Scale bar = 10 μM.

To assess the selectivity of the compounds for cancer cells, **DIPAY** and **DIPAC** were administered to normal primary (BJ) and hTERT-immortalized, large T SV40 antigen-transformed (BJEHLT) human foreskin fibroblasts. As shown in Figure 7, **DIPAC** displayed a good selectivity for BJEHLT compared to primary BJ, with **DIPAC** being more cytotoxic in the BJEHLT cell line, whereas **DIPAY**’s cytotoxic effect was comparable in the two cell lines (Figure 7A). Consistent with that, the IC_50_ dose of **DIPAC** (500 nM) triggered DDR activation, measured as the percentage of γH2AX-positive cells, in transformed but not in primary cells, confirming both the selectivity of **DIPAC** for transformed cells and the involvement of the DNA damage response pathway in the cytotoxic effect. On the contrary, **DIPAY** was not able to induce DDR in any of the cell lines analyzed, despite its elevated cytotoxicity, suggesting the involvement of other mechanisms. To finally ascertain telomeric G4s as a **DIPAC** target, we performed an immunofluorescence–FISH experiment in cells treated with an IC_50_ dose of **DIPAC**, with an anti-γH2AX-specific antibody and a cy3- telo-PNA probe to hybridize telomeric sequences. Due to the intense emission of **DIPAC** in the red channel, only U2OS cells, which possess long telomeres, displayed sufficiently intense spots of the cy3- telo-PNA probe, recognizable from the **DIPAC** fluorescence background, to allow the analysis. As shown in Figure 8, **DIPAC** treatment induced a significant increase in the TIFs per nucleus, as well as the percentage of cells displaying TIFs (Telomere’s Dysfunction-Induced Foci), corresponding to co-localizations between γH2AX foci and telomeric signals. We can thus confirm that **DIPAC** is indeed targeting telomeres, among other targets.

## 4. Conclusions

In conclusion, our study describes two novel water-soluble NDI-ceNDI dyads with a flexible and rigid linker designed to improve selectivity toward the G4 target. A full description of the fluorescent properties and the binding to ds and G4 DNA of the water-soluble NDI-ceNDI dyads has been provided. These dyads exhibit distinct optic characteristics, with potential applications in fluorescence imaging and DNA probing, thanks to their emission and visible circular dichroism features, differentiating DNA in different conformations upon binding. The spectroscopic global analysis performed allowed us to identify the most appropriate binding model and constants. Both dyads exhibit selectivity toward G4 DNA over ds DNA. The picture emerging from the analysis is that of the existence of multiple binding sites even in complexes with a unique stoichiometry. The in vitro biological evaluation further highlights their potential as therapeutic agents, with a dual functionality for both probing DNA conformation and inducing cytotoxic effects. In fact, the behavior of the two investigated dyads is quite different. Specifically, **DIPAY** exhibits a higher cytotoxicity in all the cell lines analyzed (IC_50_ values: 88 nM on U2OS and 86 nM on Hela cells), but the lack of DDR activation or selectivity for any cell type (transformed vs primary or long vs short telomeres) suggests the involvement of other possible targets and so far unexplored mechanisms of action. Contrarily, **DIPAC**, which is characterized by the presence of a more rigid and “kinked” triazole linker, exhibits lower cytotoxicity but higher specificity. Indeed, only **DIPAC** is capable of inducing DNA damage specifically in cancer cell lines, mainly targeting telomeric regions. Additionally, the telomeric targeting of DIPAC was confirmed in U2OS cells, which possess very long telomeres, by means of co-localizations between γH2AX foci and telomeric-specific antibody signals. Overall, our findings comparing two dyads featuring a different linker confirm the importance of ligand design to optimize target selectivity, and they provide valuable insights for the future development of DIPAC as a novel theragnostic agent. 

## Figures and Tables

**Table 1:** Fluorescence quantum yields and fluorescence lifetimes of the three new compounds, **PHAM-Prop**, **DIPAY** and **DIPAC**, in water with or without SDS.

Sample	Φ ^a^	Lifetime, τ (ns),^b^ Measured at 605 nm		
		λ_exc_ 560 nm	λ_exc_ 465 nm	
		τ_1_	τ_1_	τ_2_
**PHAM-Prop**	0.10	3.34	3.23	
**PHAM-Prop** **/SDS**	0.32	3.94	3.80	
**DIPAY**	0.004	3.35	2.79 (59%)	7.32 (41%)
**DIPAY/SDS**	0.32	3.71	3.71	
**DIPAC**	<0.001	3.31	2.65 (22%)	8.80 (78%)
**DIPAC/SDS**	0.053	3.47	3.44	

^a^ Using **NDI-tri-propyl** (Chart S1) as a reference with a value of 0.17 in phosphate buffer of pH 2; error of 10% [29]. All solutions are isosbestic at the 441 nm excitation wavelength. ^b^ Statistical error of ±0.01 ns for the lifetime; the relative amplitude in percentage is reported in the case of fitting with the bi-exponential function.

**Table 2:** Complex (DNA/dyad) stoichiometry and logK of binding constants obtained upon global analysis of fluorescence spectra in Figure 2 and Figure S4.

DNA	PHAM-Prop		DIPAY		DIPAC	
**Htel22**	1:1 1:2	7.64 ± 0.16 14.04 ± 0.21	1:1 1:2 2:1	8.93 ± 0.07 15.71 ± 0.10 14.14 ± 0.06	1:1 1:2 1:3	7.67 ± 0.05 14.68 ± 0.10 20.37 ± 0.10
**Htel45**	1:2 1:4	14.41 ± 0.05 28.6 ± 0.1	1:1 1:2 1:4 2:1	7.91 ± 0.07 12.94 ± 0.16 25.52 ± 0.07 13.24 ± 0.08	1:2 1:4 2:1	13.80 ± 0.05 26.88 ± 0.1 11.55 ± 0.03
**c-myc22**	1:1 1:2	6.89 ± 0.05 14.74 ± 0.06	1:1 1:2	6.16 ± 0.02 13.78 ± 0.03	1:1 1:2 2:1	7.77 ± 0.12 14.36 ± 0.16 11.69 ± 0.16
**Ds26**	1:2 1:4	15.25 ± 0.03 29.15 ± 0.04	1:1 1:2 1:3	6.50 ± 0.03 12.95 ± 0.03 18.35 ± 0.05	1:1 1:2 1:3	5.82 ± 0.02 12.48 ± 0.02 17.70 ± 0.12

**Table 3:** Fluorescence lifetime and relative amplitudes α_j_ obtained from bi-exponential fitting in the presence of excess DNA, and the main complexes present in solution.

	τ_1_, ns	α_1_	τ_2_, ns	α_2_	<τ_av_>/ns	DNA/Ligand
**PHAM-Prop**						
**hTel22**	1.25	62%	3.22	38%	2.45	1:1
**hTel45**	1.00	55%	3.5	45%	2.85	1:2
**Cmyc22**	0.68	66%	3.15	34%	2.42	1:1, 1:2
**Ds26mer**	0.39	89%	3.14	11%	1.76	1:2
**DIPAY**						
**hTel22**	0.77	69%	2.77	31%	2.00	1:1, 2:1
**hTel45**	0.68	83%	2.52	17%	1.47	1:1, 2:1
**Cmyc22**	0.47	88%	1.70	12%	0.88	1:1, 1:2
**Ds26mer**	0.20	90%	0.68	10%	0.33	1:1, 1:2
**DIPAC**						
**hTel22**	0.74	74%	2.53	26%	1.72	1:1, 1:2
**hTel45**	0.90	73%	2.44	27%	1.67	1:1, 2:1
**Cmyc22**	0.52	85%	1.82	15%	1.01	1:1, 2:1
**Ds26mer**	0.19	90%	1.37	10%	0.71	1:1, 1:2

Statistical error of ±0.01 ns for the lifetime; α_j_ represents the relative amplitudes, i.e., fractional pre-exponential factors, obtained upon fitting with the bi-exponential function.

## Data Availability

The data supporting this article have been included as part of the Supplementary Information.

## Supplementary Materials

The following supporting information can be downloaded at: https://www.mdpi.com/article/10.3390/biom15020311/s1.
