# Supplementary material for: Core-Extended Naphthalene Diimide Dyads as Light-Up Probes with Targeted Cytotoxicity Toward Tumor Cells
Source: Biomolecules. 2025 Feb 19;15(2):311. doi: 10.3390/biom15020311 (PMC11852925; doi:10.3390/biom15020311)

Supporting Information

Core-extended Naphthalene Diimide dyads as light-up probes with targeted cytotoxicity toward tumor cells

Valentina Pirota,[1] Erica Salvati,[2] Carla Risoldi,[2] Francesco Manoli,[3] Angela Rizzo,[4] Pasquale Zizza,[4] Annamaria Biroccio,[4] Mauro Freccero,[1] Ilse Manet,[3,*], and Filippo Doria,[1,*]

1 Dipartimento di Chimica, Università di Pavia. V.le Taramelli 10, 27100 Pavia, Italy.
2 Institute of Molecular Biology and Pathology, Consiglio Nazionale delle Ricerche, Via degli Apuli 4, 00185 Roma, Italy
3 Istituto per la Sintesi Organica e la Fotoreattività, Consiglio Nazionale delle Ricerche, via Gobetti 101, 40129 Bologna, Italy.
4 Translational Oncology Research Unit, IRCCS-Regina Elena National Cancer Institute, Via Elio Chianesi 53, 00144 Rome, Italy.
* Correspondence: filippo.doria@unipv.it; ilse.manet@isof.cnr.it

Chart S1: **NDI-tri-propyl** and NDI-dimer reference compound of the photophysical study

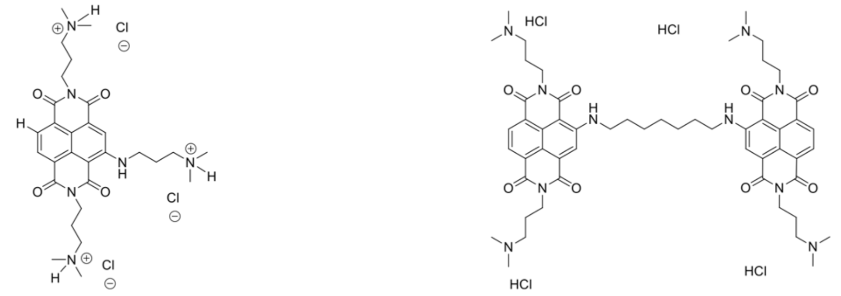

Figure S1: Absorption spectra (a), fluorescence spectra (b) and normalized excitation spectra (c-e) of 5 µM solutions of **PHAM-Prop, DIPAY** and **DIPAC,** with and without SDS**;** graphs c-e report also the normalized absorption spectra for the sake of comparison. For the sake of comparison also the absorption spectrum of **NDI-tri-propyl (NDI)** is shown.

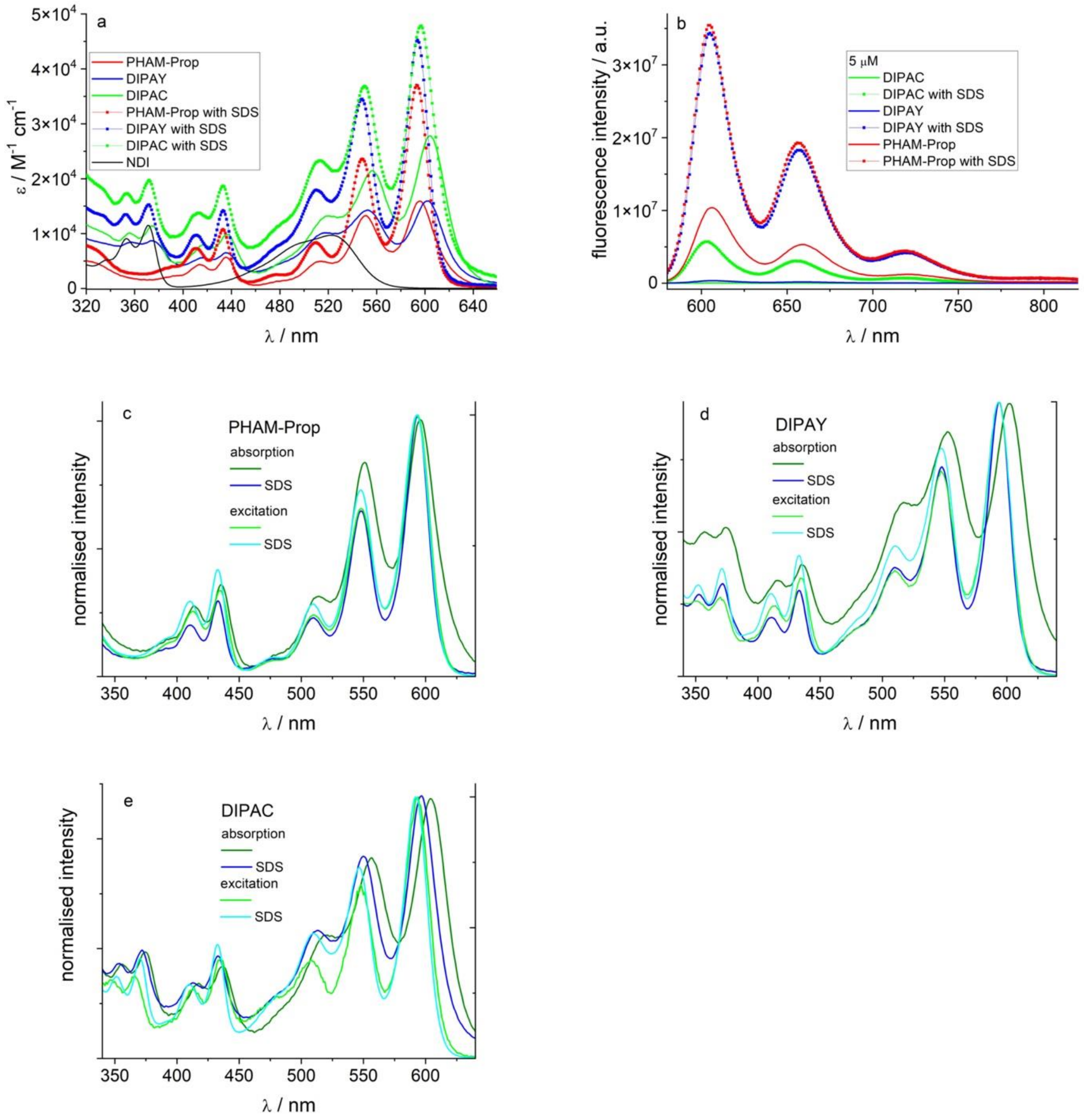

Figure S2: dilution experiment in 10 mM phosphate buffer with 100 mM K^+, conditions similar to the titration study. Left column: the absorption spectra of PHAM-prop, DIPAY and DIPAC at different concentrations (optical path: 1 cm); central panel: molar absorption coefficient spectra of most diluted and concentrated solutions of PHAM-prop, DIPAY and DIPAC (optical path: 1 cm); right column represents the calculated spectra of the PHAM-prop monomer and dimer obtained with the dimerization constant logK 6,6.

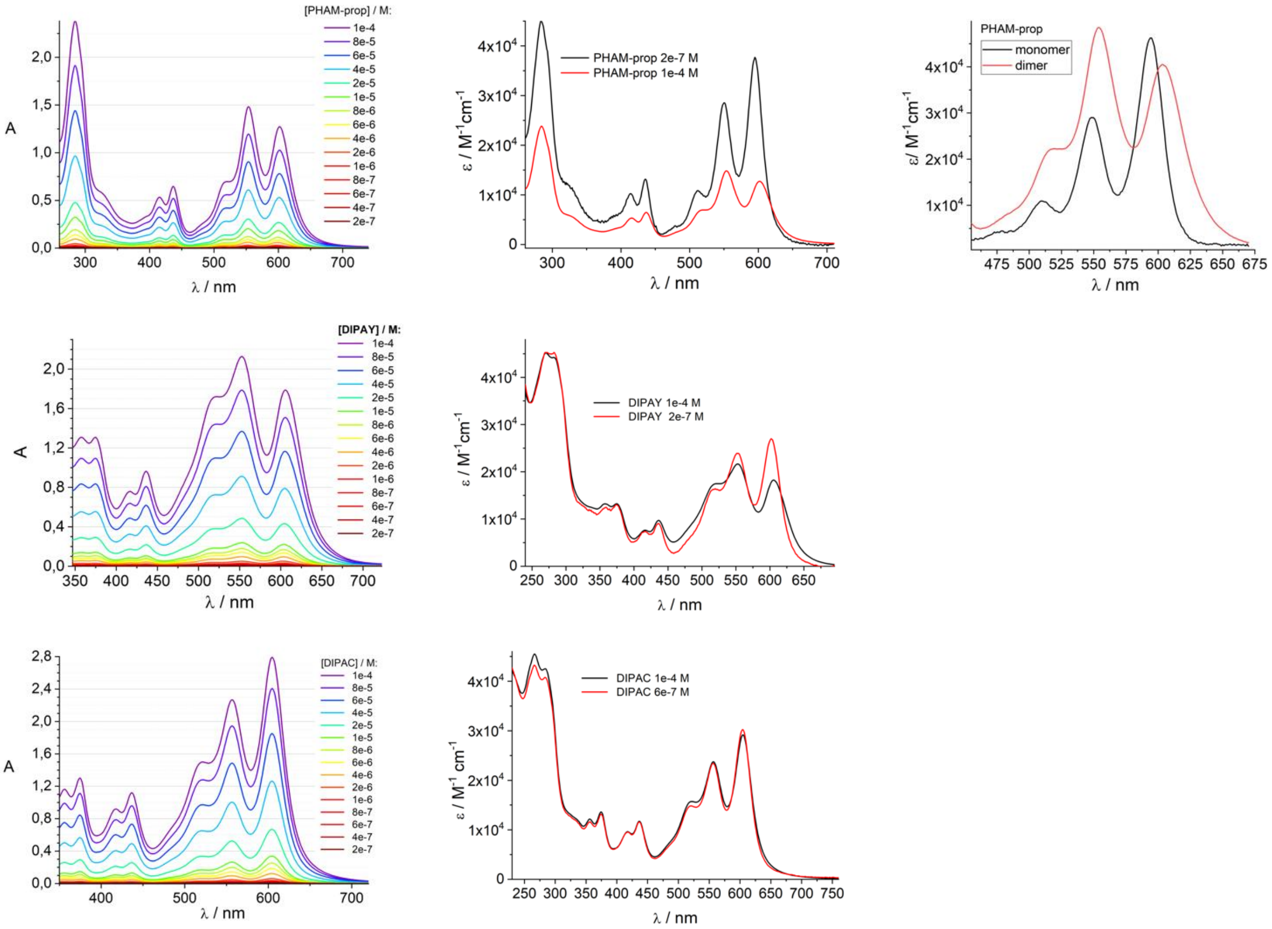

Table S1a: absorbances of the peaks at 550 nm (A1) and 595 nm (A2) in water with and without SDS and for the lowest diluted solutions:

	λ / nm	A1	λ / nm	A2	A2/A1
PhAM-Prop	551	0,073	596	0,087	1,19
PhAM-Prop SDS	548	0,13	593	0,203	1,56[a]
0.2 µM PhAM-Prop					1,31
DIPAY	552	0,06	602	0,067	1,12
DIPAY SDS	548	0,145	594	0,19	1,31[b]
0.2 µM DIPAY					1,13
DIPAC	556	0,053	604	0,069	1,30
DIPAC SDS	550	0,092	597	0,119	1,29
0.4 µMDIPAC					1,29[a]

a Note the excitation spectrum of mPAP and dPAC in water have a ratio of 1.54 and 1.56.
b Note the excitation spectrum of dPAY in water has the same ratio of 1.33.

Table S1b: Molar absorption coefficients of 5 µM solutions:

	λ / nm	ε / $M^{-1}cm^{-1}$	λ / nm	ε / $M^{-1}cm^{-1}$	ratio
**1, PHAM-Prop**	551	13260	596	15890	1,19
**1, PHAM-Prop SDS**	548	23600	594	36900	1,56
**2, DIPAY**	552	14280	603	15930	1,11
**2, DIPAY SDS**	548	34500	594	45200	1,31
**3, DIPAC**	557	21500	604	27840	1,29
**3, DIPAC SDS**	550	36800	597	47800	1,30

DNA binding study

Figure S3: absorption spectra of PHAM-Prop, DIPAY and DIPAC with increasing amounts of DNA; 1 cm cuvette

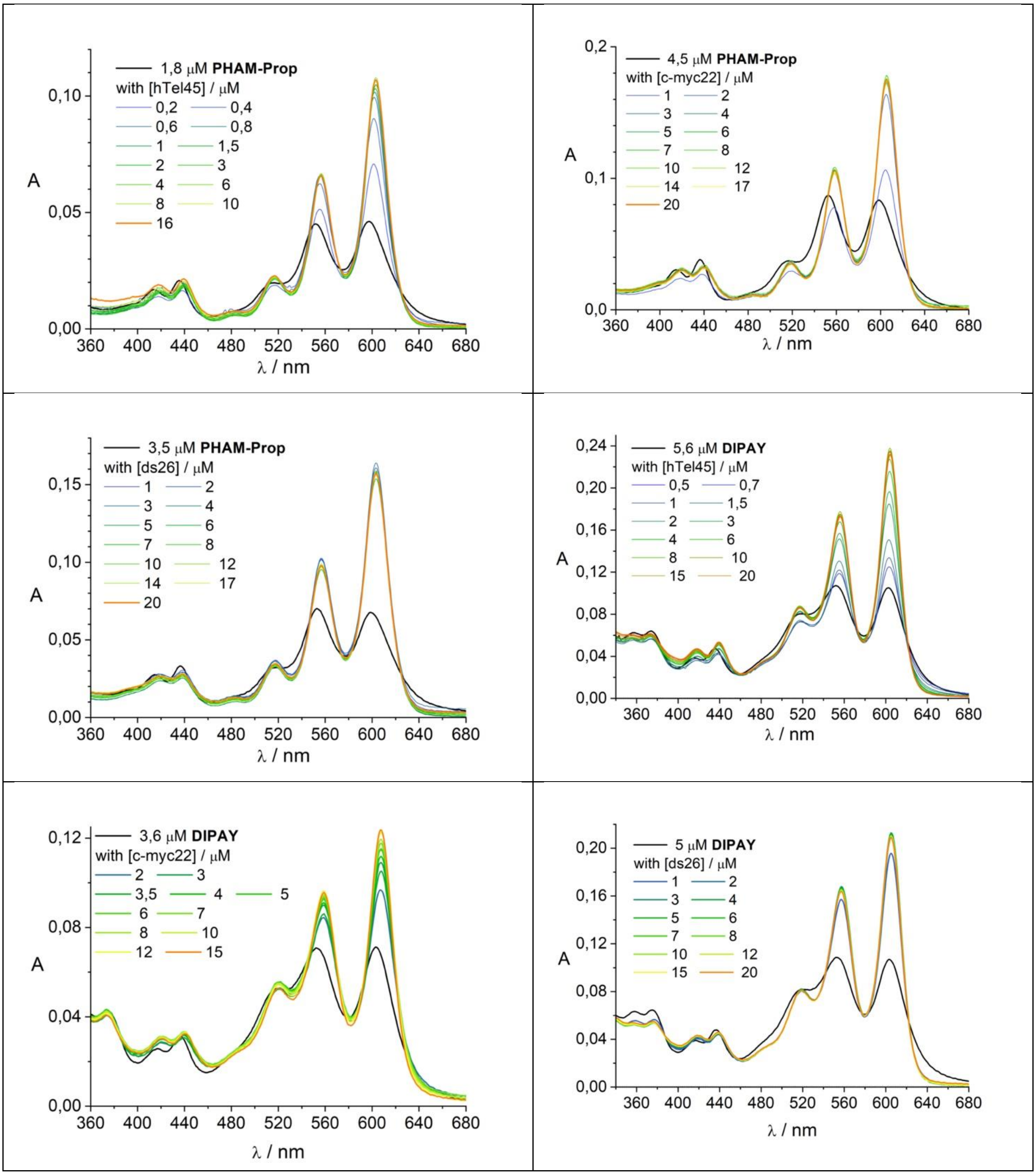

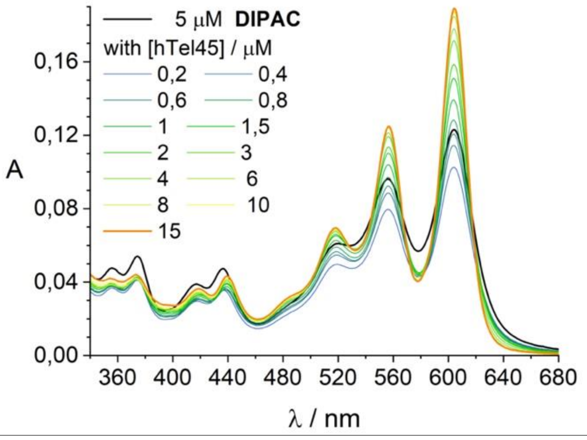
5 μM DIPAC
with [hTel45] / μM
0,2
0,4
0,6
0,8
1
1,5
2
3
4
6
8
10
15
A
0,00
0,04
0,08
0,12
0,16
360 400 440 480 520 560 600 640 680
λ / nm

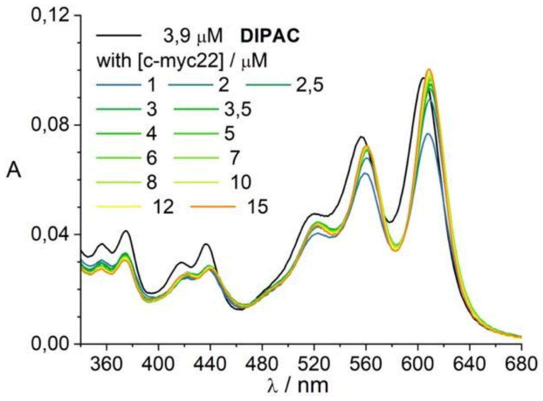
3,9 μM DIPAC
with [c-myc22] / μM
1
2
2,5
3
3,5
4
5
6
7
8
10
12
15
A
0,00
0,04
0,08
0,12
360 400 440 480 520 560 600 640 680
λ / nm

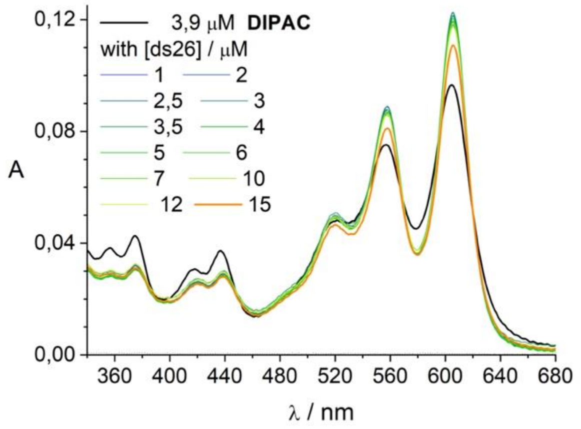
3,9 μM DIPAC
with [ds26] / μM
1
2
2,5
3
3,5
4
5
6
7
10
12
15
A
0,00
0,04
0,08
0,12
360 400 440 480 520 560 600 640 680
λ / nm

Figure S4: Fluorescence spectra of PHAM-Prop, DIPAY and DIPAC with increasing amounts of DNA

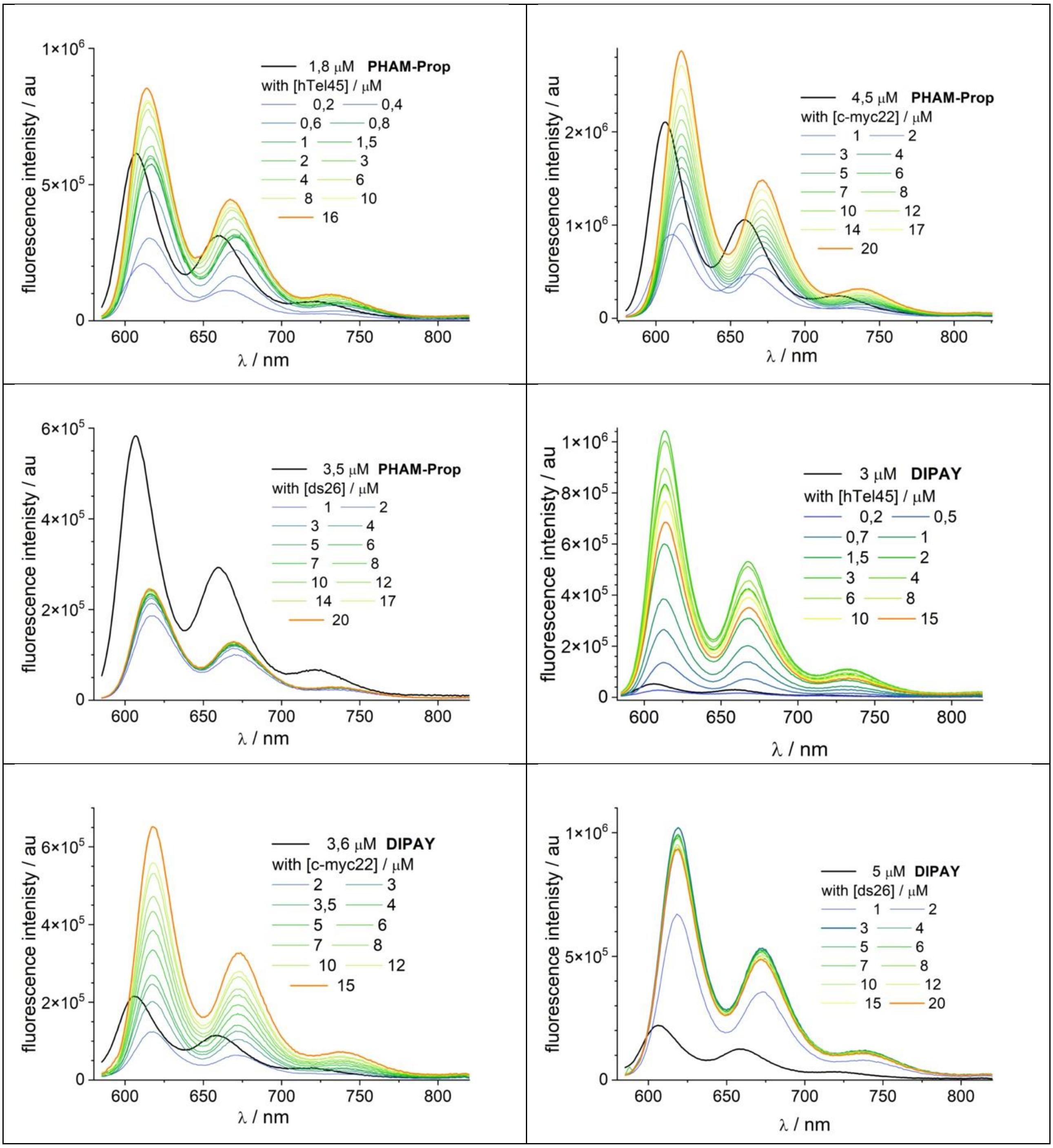

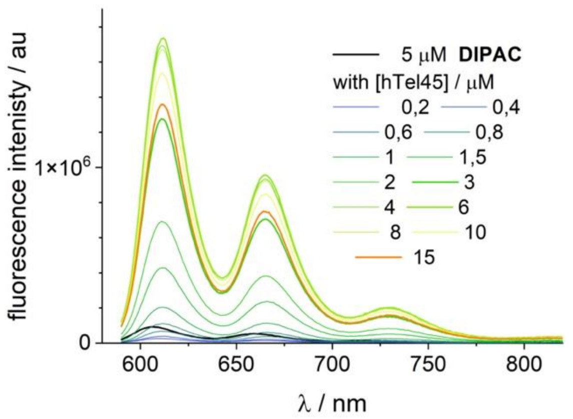

fluorescence intenisty / au
1×10^6
0
600
650
700
750
800
λ / nm
5 μM DIPAC
with [hTel45] / μM
0,2
0,4
0,6
0,8
1
1,5
2
3
4
6
8
10
15

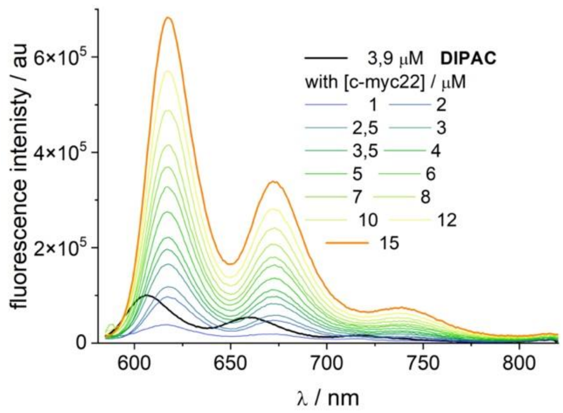

fluorescence intenisty / au
6×10^5
4×10^5
2×10^5
0
600
650
700
750
800
λ / nm
3,9 μM DIPAC
with [c-myc22] / μM
1
2
2,5
3
3,5
4
5
6
7
8
10
12
15

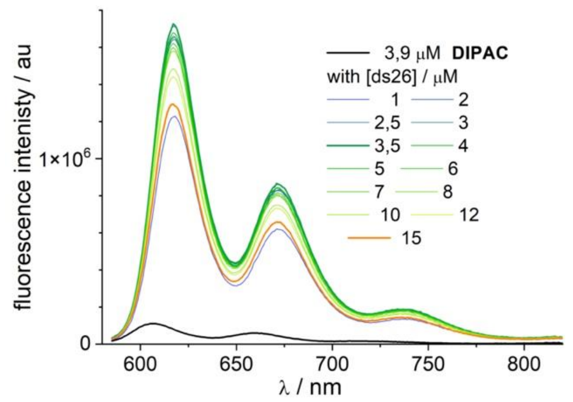

fluorescence intenisty / au
1×10^6
0
600
650
700
750
800
λ / nm
3,9 μM DIPAC
with [ds26] / μM
1
2
2,5
3
3,5
4
5
6
7
8
10
12
15

Figure S5: Circular dichroism spectra, normalized in the UV range, and original ellipticity in the visible in 2 cm cuvet for PHAM-Prop, DIPAY and DIPAC with increasing amounts of DNA.

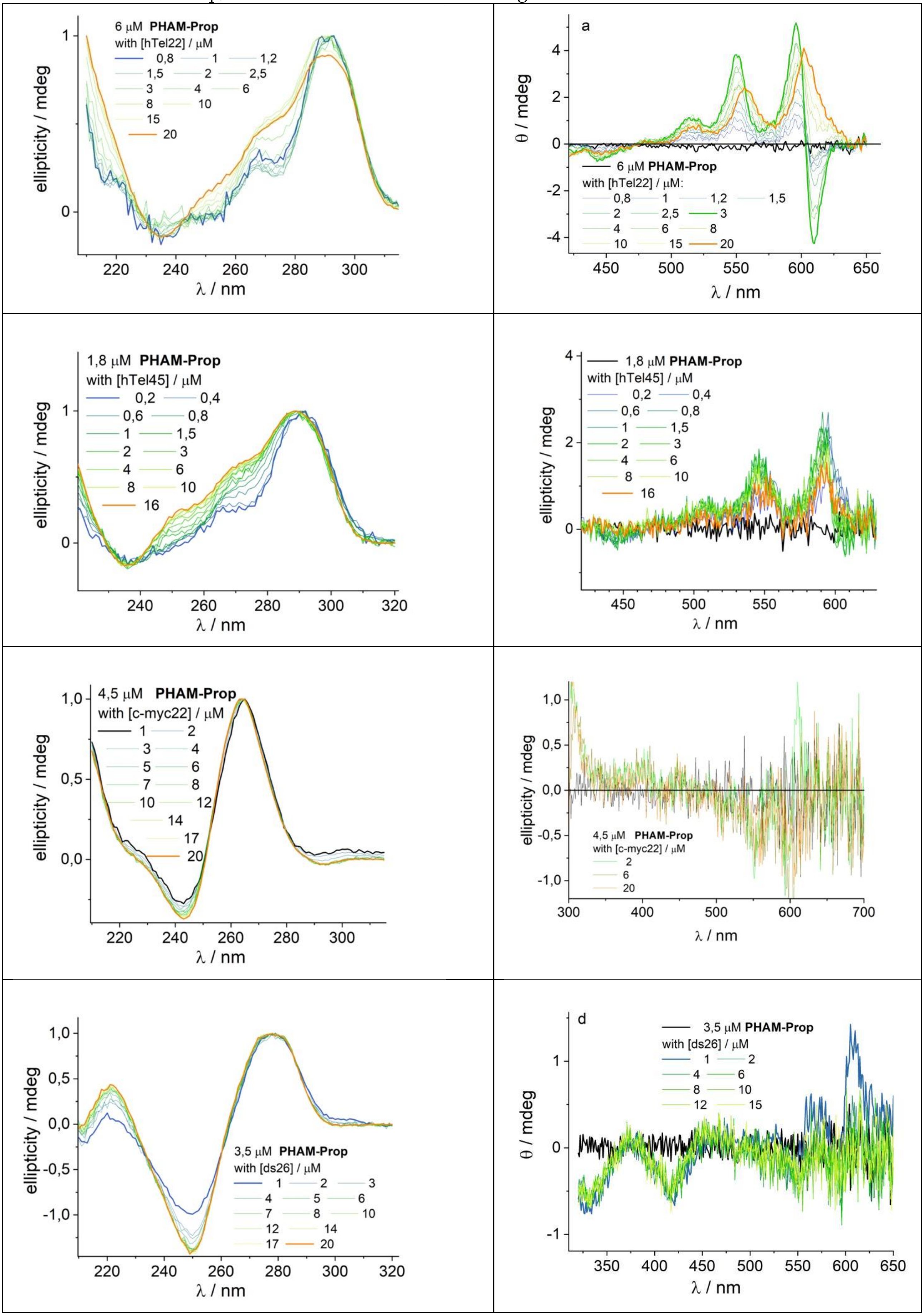

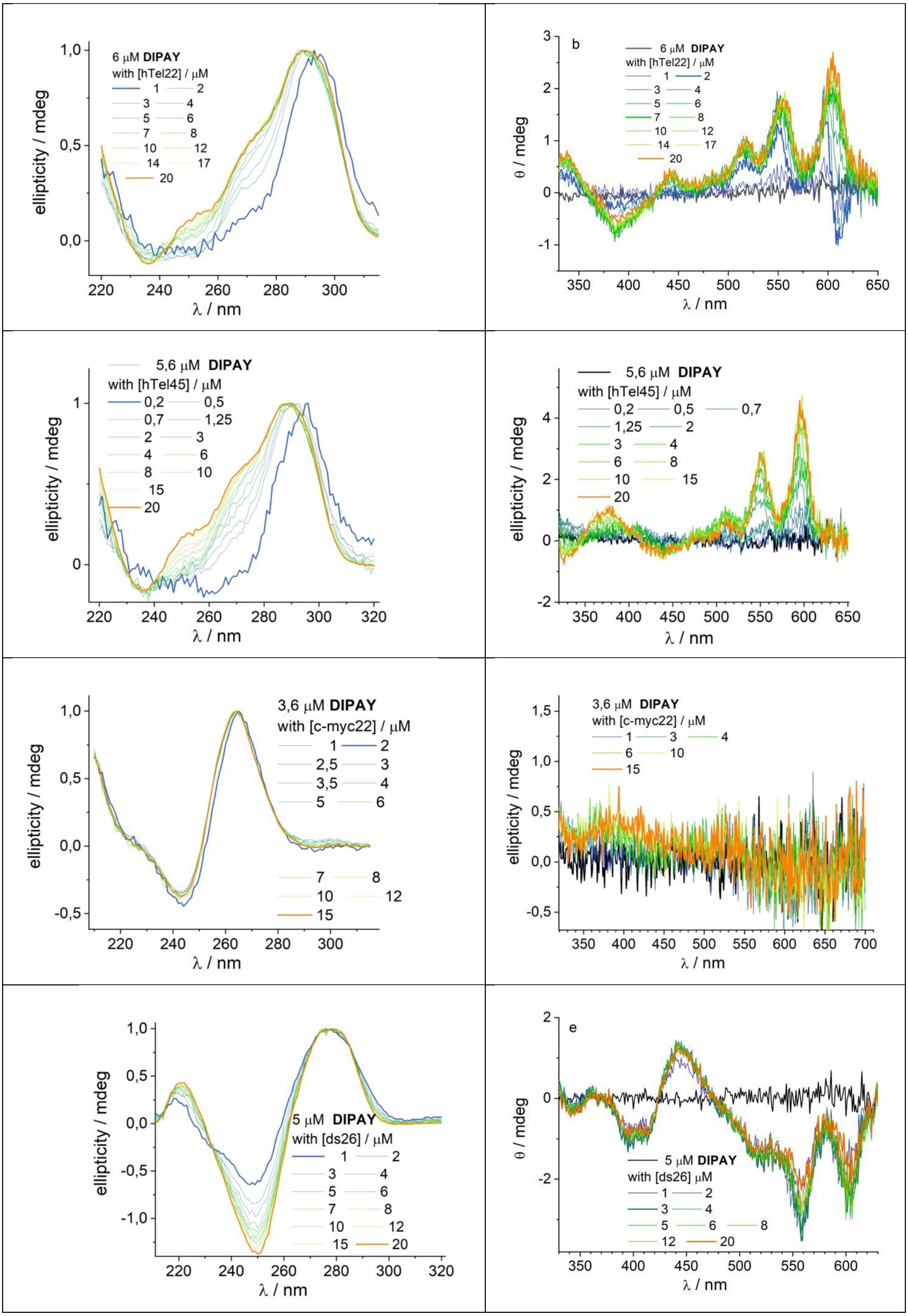
6 μM DIPAY
with [hTel22] / μM
1 2 3 4 5 6 7 8 10 12 14 17 20
ellipticity / mdeg
λ / nm
b
6 μM DIPAY
with [hTel22] / μM
1 2 3 4 5 6 7 8 10 12 14 17 20
θ / mdeg
λ / nm
5,6 μM DIPAY
with [hTel45] / μM
0,2 0,5 0,7 1,25 2 3 4 6 8 10 15 20
ellipticity / mdeg
λ / nm
5,6 μM DIPAY
with [hTel45] / μM
0,2 0,5 0,7 1,25 2 3 4 6 8 10 15 20
ellipticity / mdeg
λ / nm
3,6 μM DIPAY
with [c-myc22] / μM
1 2 2,5 3 3,5 4 5 6 7 8 10 12 15
ellipticity / mdeg
λ / nm
3,6 μM DIPAY
with [c-myc22] / μM
1 3 4 6 10 15
ellipticity / mdeg
λ / nm
5 μM DIPAY
with [ds26] / μM
1 2 3 4 5 6 7 8 10 12 15 20
ellipticity / mdeg
λ / nm
e
5 μM DIPAY
with [ds26] μM
1 2 3 4 5 6 8 12 20
θ / mdeg
λ / nm

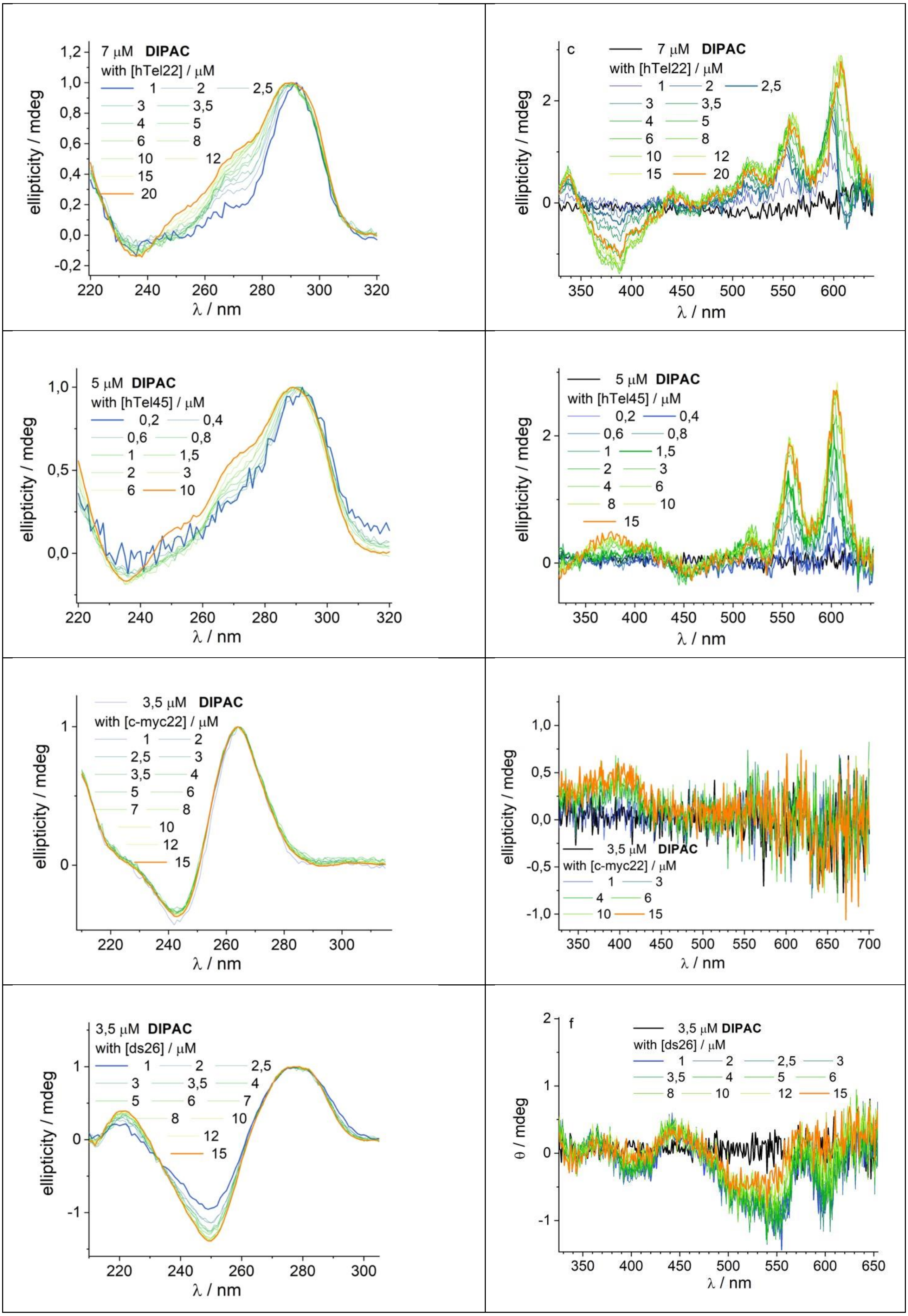
7 μM DIPAC
with [hTel22] / μM
1 2 2,5 3 3,5 4 5 6 8 10 12 15 20
ellipticity / mdeg
λ / nm
c
7 μM DIPAC
with [hTel22] / μM
1 2 2,5 3 3,5 4 5 6 8 10 12 15 20
ellipticity / mdeg
λ / nm
5 μM DIPAC
with [hTel45] / μM
0,2 0,4 0,6 0,8 1 1,5 2 3 6 10
ellipticity / mdeg
λ / nm
5 μM DIPAC
with [hTel45] / μM
0,2 0,4 0,6 0,8 1 1,5 2 3 4 6 8 10 15
ellipticity / mdeg
λ / nm
3,5 μM DIPAC
with [c-myc22] / μM
1 2 2,5 3 3,5 4 5 6 7 8 10 12 15
ellipticity / mdeg
λ / nm
3,5 μM DIPAC
with [c-myc22] / μM
1 3 4 6 10 15
ellipticity / mdeg
λ / nm
3,5 μM DIPAC
with [ds26] / μM
1 2 2,5 3 3,5 4 5 6 7 8 10 12 15
ellipticity / mdeg
λ / nm
f
3,5 μM DIPAC
with [ds26] / μM
1 2 2,5 3 3,5 4 5 6 8 10 12 15
θ / mdeg
λ / nm

Figure S6: calculated fluorescence spectra (left); species concentration plot (middle) and α_j (alfa) values plot vs total NA concentration (right).

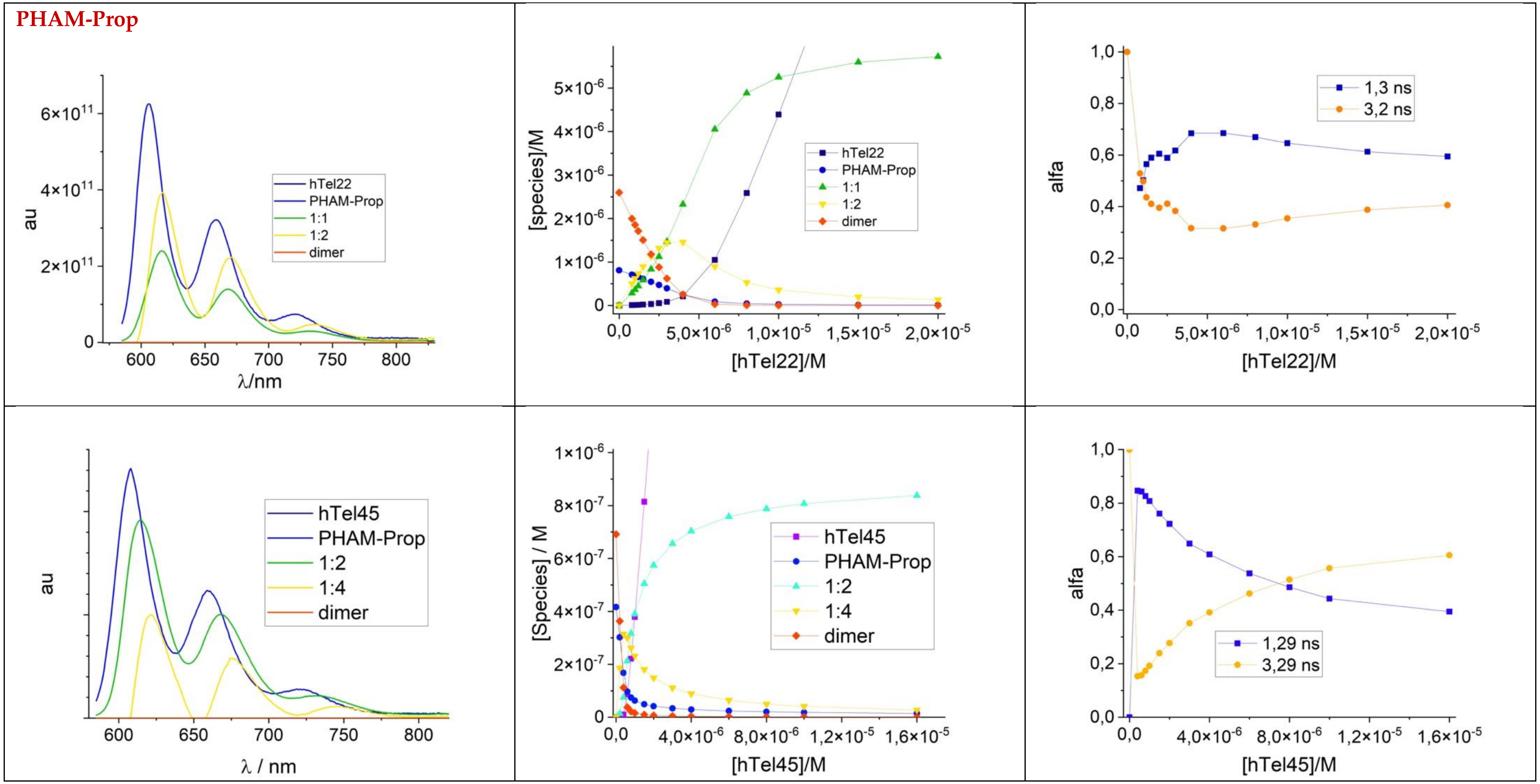

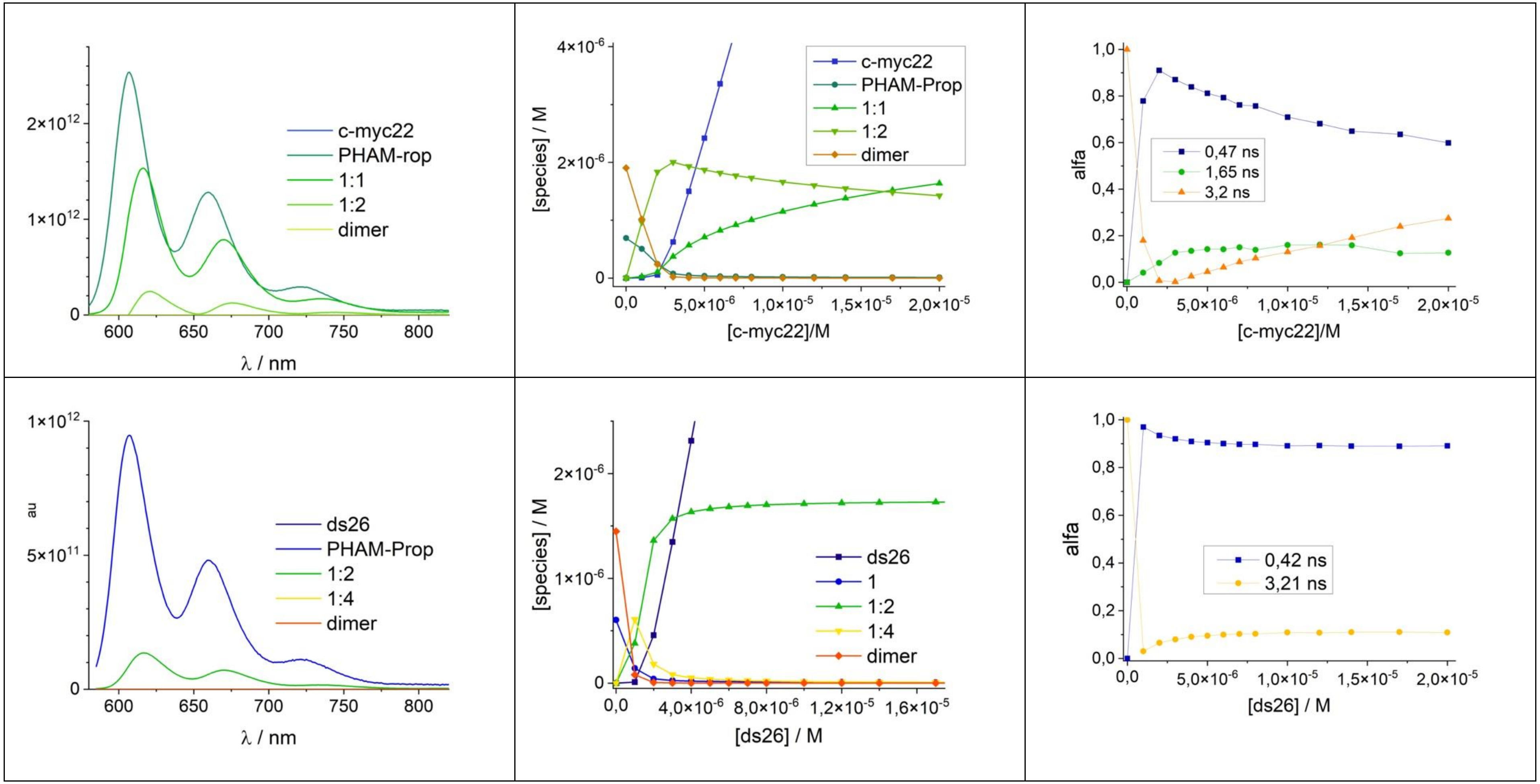
2×10^12
1×10^12
0
600 650 700 750 800
λ / nm
c-myc22
PHAM-rop
1:1
1:2
dimer
4×10^-6
2×10^-6
0
[species] / M
0,0 5,0×10^-6 1,0×10^-5 1,5×10^-5 2,0×10^-5
[c-myc22]/M
c-myc22
PHAM-Prop
1:1
1:2
dimer
1,0 0,8 0,6 0,4 0,2 0,0
alfa
0,47 ns
1,65 ns
3,2 ns
0,0 5,0×10^-6 1,0×10^-5 1,5×10^-5 2,0×10^-5
[c-myc22]/M
1×10^12
5×10^11
0
au
600 650 700 750 800
λ / nm
ds26
PHAM-Prop
1:2
1:4
dimer
2×10^-6
1×10^-6
0
[species] / M
0,0 4,0×10^-6 8,0×10^-6 1,2×10^-5 1,6×10^-5
[ds26] / M
ds26
1
1:2
1:4
dimer
1,0 0,8 0,6 0,4 0,2 0,0
alfa
0,42 ns
3,21 ns
0,0 5,0×10^-6 1,0×10^-5 1,5×10^-5 2,0×10^-5
[ds26] / M

DIPAY

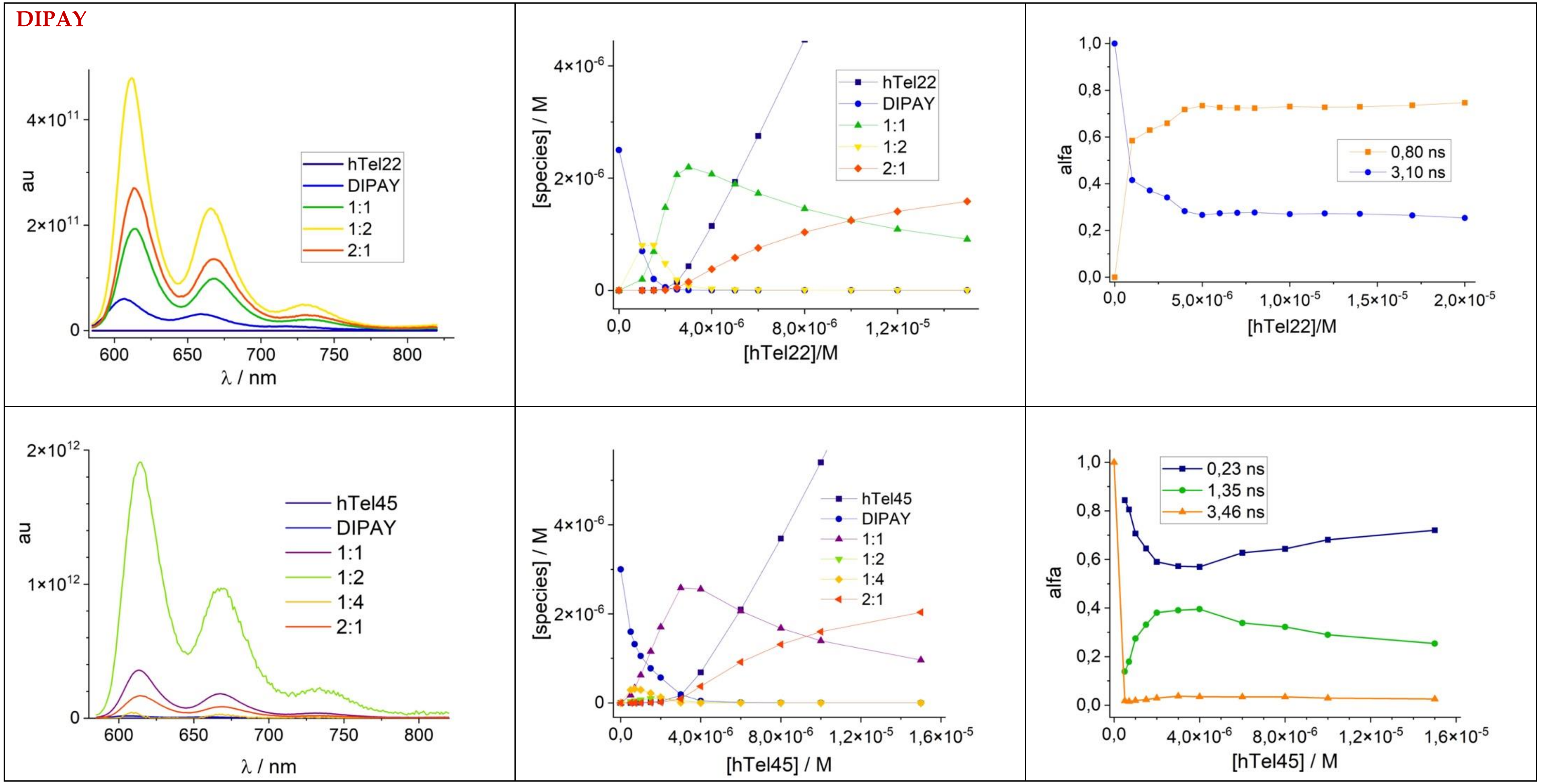

au
λ / nm
hTel22
DIPAY
1:1
1:2
2:1
[species] / M
[hTel22]/M
alfa
0,80 ns
3,10 ns
hTel45
1:4
[hTel45] / M
0,23 ns
1,35 ns
3,46 ns

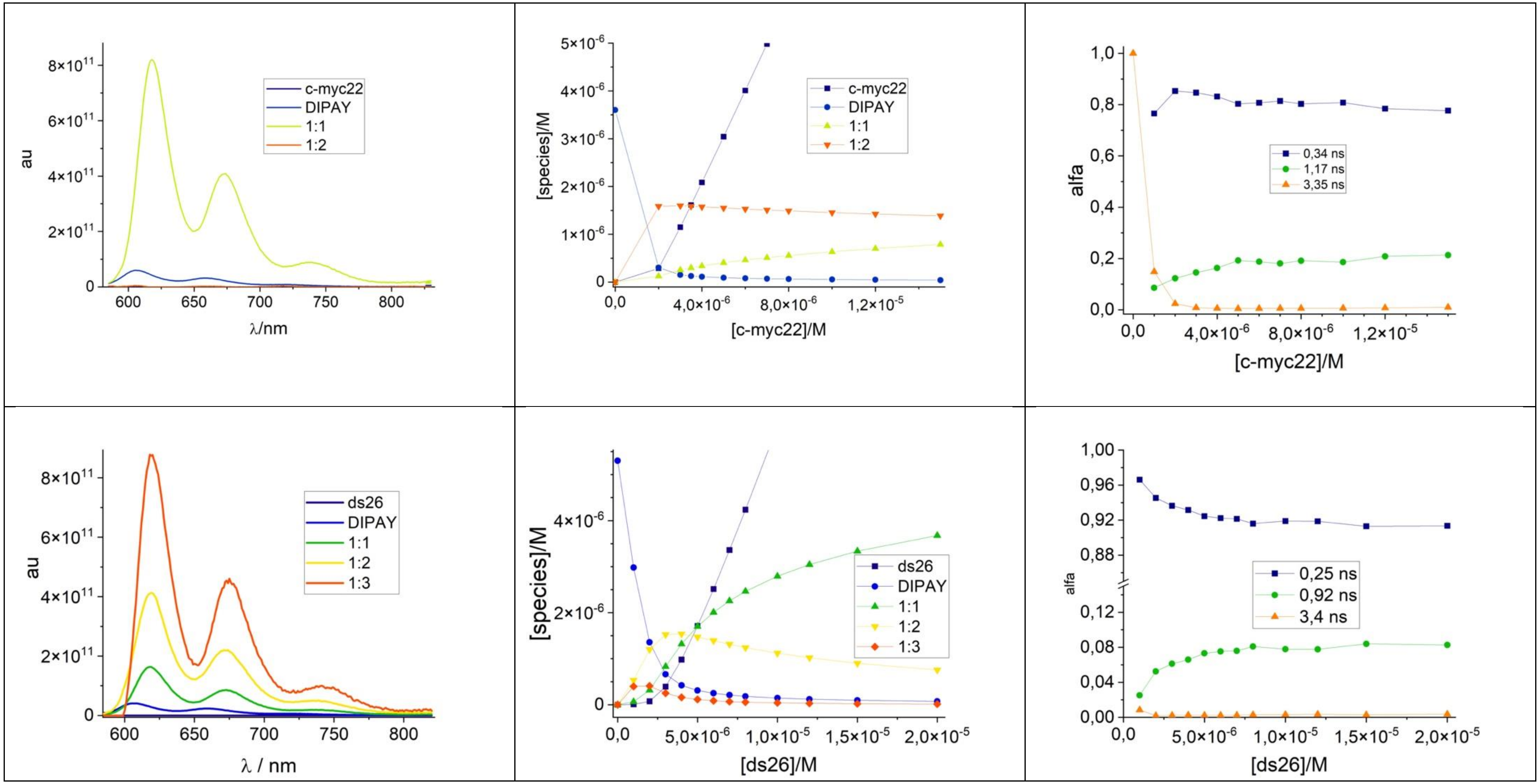
8×10^11
6×10^11
4×10^11
2×10^11
0
au
600 650 700 750 800
λ/nm
c-myc22
DIPAY
1:1
1:2
5×10^-6
4×10^-6
3×10^-6
2×10^-6
1×10^-6
0
[species]/M
0,0 4,0×10^-6 8,0×10^-6 1,2×10^-5
[c-myc22]/M
c-myc22
DIPAY
1:1
1:2
1,0
0,8
0,6
0,4
0,2
0,0
alfa
0,0 4,0×10^-6 8,0×10^-6 1,2×10^-5
[c-myc22]/M
0,34 ns
1,17 ns
3,35 ns
8×10^11
6×10^11
4×10^11
2×10^11
0
au
600 650 700 750 800
λ / nm
ds26
DIPAY
1:1
1:2
1:3
4×10^-6
2×10^-6
0
[species]/M
0,0 5,0×10^-6 1,0×10^-5 1,5×10^-5 2,0×10^-5
[ds26]/M
ds26
DIPAY
1:1
1:2
1:3
1,00
0,96
0,92
0,88
0,12
0,08
0,04
0,00
alfa
0,0 5,0×10^-6 1,0×10^-5 1,5×10^-5 2,0×10^-5
[ds26]/M
0,25 ns
0,92 ns
3,4 ns

DIPAC

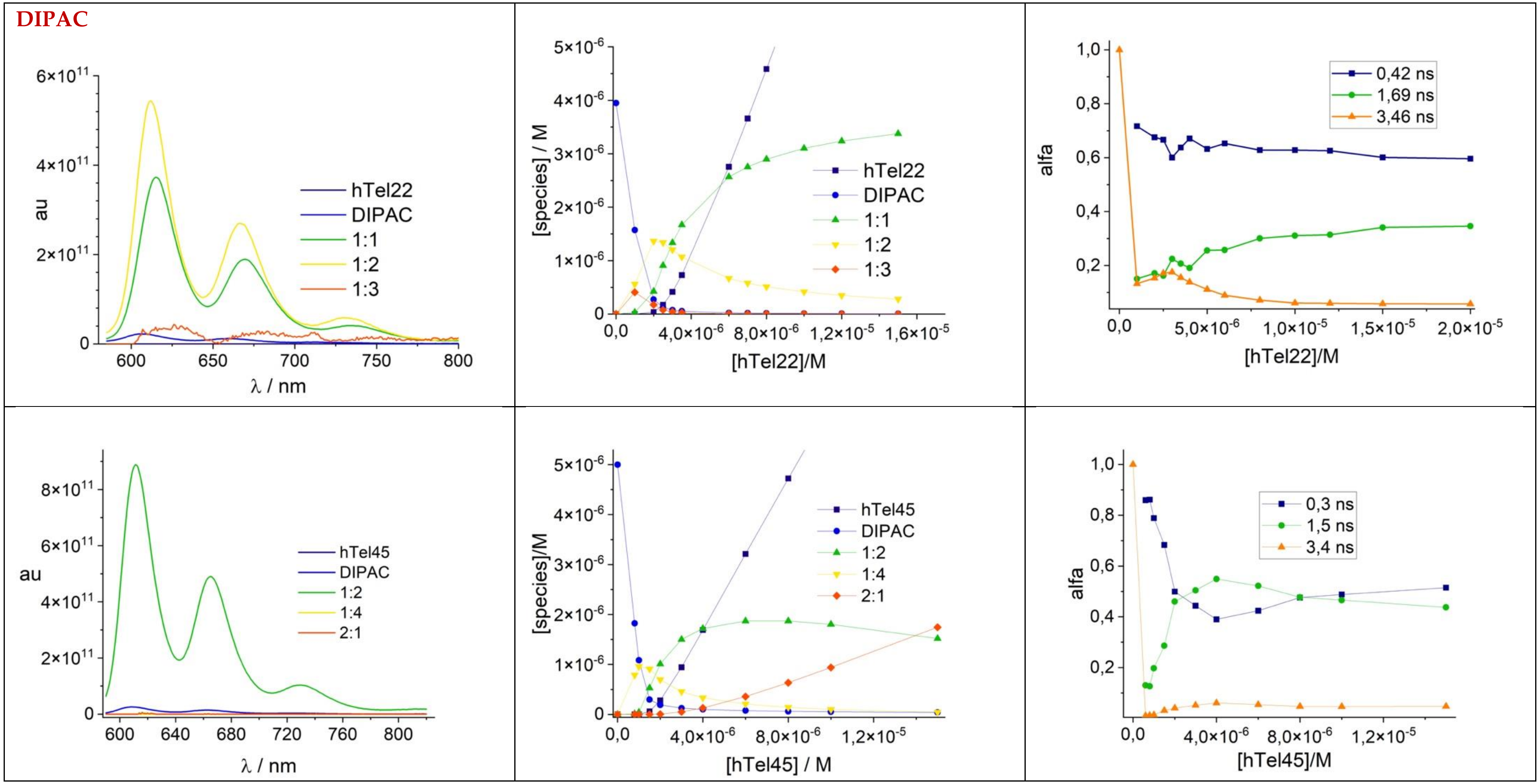
au
λ / nm
hTel22
DIPAC
1:1
1:2
1:3
[species] / M
[hTel22]/M
alfa
0,42 ns
1,69 ns
3,46 ns
hTel45
1:4
2:1
[species]/M
[hTel45] / M
[hTel45]/M
0,3 ns
1,5 ns
3,4 ns

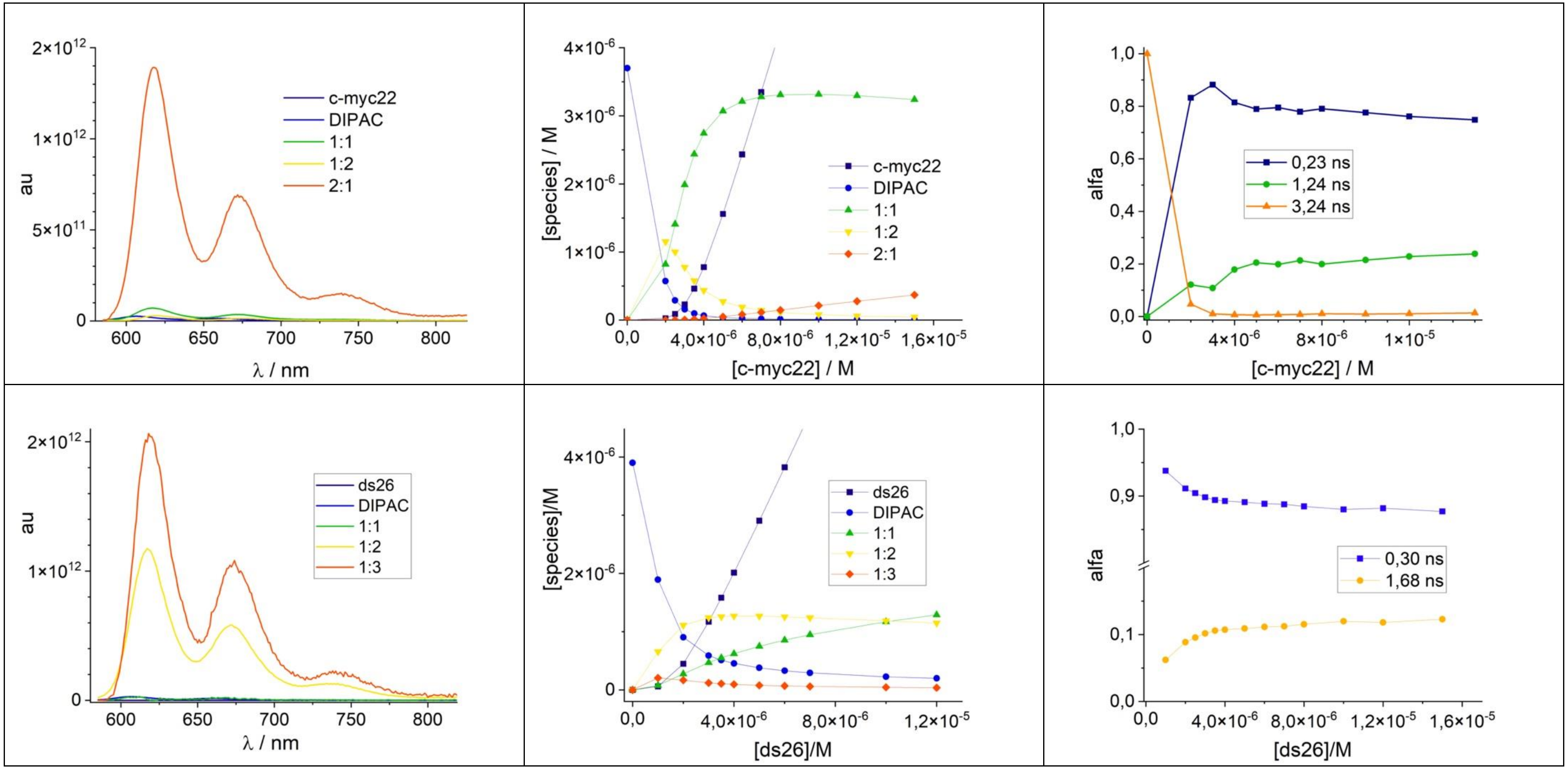

au
λ / nm
c-myc22
DIPAC
1:1
1:2
2:1
[species] / M
[c-myc22] / M
alfa
0,23 ns
1,24 ns
3,24 ns
ds26
DIPAC
1:1
1:2
1:3
[species]/M
[ds26]/M
0,30 ns
1,68 ns

Tables S2. Global analysis fitting parameters for DIPAY and hTEL22 with τ_1 = 0.80 ns and τ_2 = 3.10 ns

[DNA]/μM	a_1	a_2	χ^2	τ_{av_int}/ns
0	-0.029	0.064	1.3	3.40
1	0.041	0.029	1.1	2.49
2	0.049	0.029	1.3	2.40
3	0.049	0.026	1.2	2.33
4	0.058	0.023	0.95	2.19
5	0.062	0.022	1.0	2.14
6	0.059	0.022	1.0	2.16
7	0.058	0.022	1.0	2.17
8	0.058	0.022	1.1	2.17
10	0.059	0.022	1.0	2.15
12	0.060	0.022	1.0	2.16
14	0.061	0.023	1.0	2.16
17	0.059	0.021	0.74	2.14
20	0.059	0.020	0.85	2.11

Global analysis fitting parameters for DIPAY and c-myc22 with τ_1 = 0.33 ns, τ_2 = 1.15 ns and τ_3 = 3.19 ns

[DNA]/ μM	a_1	a_2	a_3	χ^2	τ_{av_int}/ns
0	-0.006	-0.005	0.058	1.0	3.303
1	0.104	0.006	0.021	1.0	2.152
2	0.139	0.019	0.004	1.1	1.034
3	0.138	0.024	0.001	1.0	0.791
4	0.130	0.026	0.001	0.8	0.766
5	0.118	0.028	0.001	0.7	0.786
6	0.129	0.030	0.001	0.7	0.792
7	0.130	0.029	0.001	0.7	0.787
8	0.122	0.029	0.001	0.7	0.803
10	0.130	0.030	0.001	0.6	0.803
12	0.119	0.032	0.001	0.7	0.846
15	0.116	0.032	0.002	0.7	0.887

Global analysis fitting parameters for DIPAY and ds26 with τ_1 = 0.26 ns , τ_2 = 1.01 ns and τ_3 = 3.19 ns

[DNA]/ μM	a_1	a_2	a_3	χ^2	τ_{av_int}/ns
0	-0.007	0.001	0.051	1.0	3.206
1	0.232	0.005	-7.26E-4	1.2	0.208
2	0.223	0.008	-9.58E-4	1.0	0.219
3	0.224	0.013	-9.31E-4	1.0	0.269
4	0.219	0.014	-6.96E-4	1.1	0.312
5	0.213	0.013	-6.92E-4	1.1	0.312
6	0.208	0.012	-5.28E-4	1.0	0.326
8	0.214	0.014	-6.01E-4	1.0	0.326
10	0.213	0.013	-5.12E-4	1.1	0.336
12	0.208	0.014	-3.14E-4	1.0	0.37
15	0.208	0.014	-4.03E-4	1.1	0.363
20	0.206	0.015	-3.48E-4	1.1	0.375

Figure S7. DDR activation in U2OS and Hela cells: Hela and U2OS were seeded and treated as previously reported, then fixed and processed for IF analysis with the rabbit anti-53BP1 antibody ant the mouse anti-phospho γH2AX antibody. The percentage of 53BP1 foci, colocalizing with γH2AX foci, is reported in the histograms. The mean of three independent experiments is shown, bars are SD. Statistically significant differences were calculated by Student's t-test, *=P<0.05. Representative images of Hela cells processed as described are given in the right panel. Scalebar =10μm.

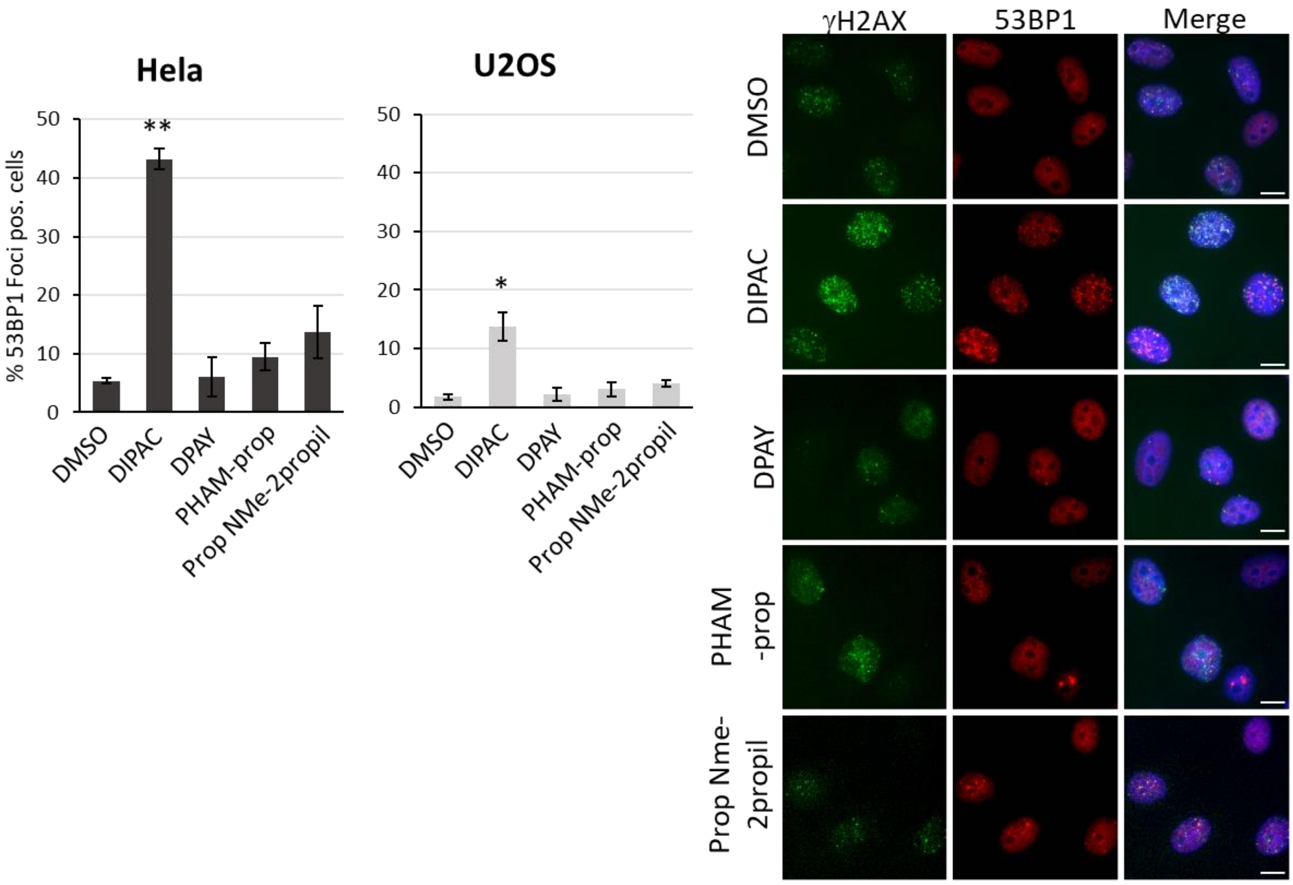

HPLC data (λ = 254 nm)

PropNMe2Propyl. (Rt = 5.83 min; 99.50%)

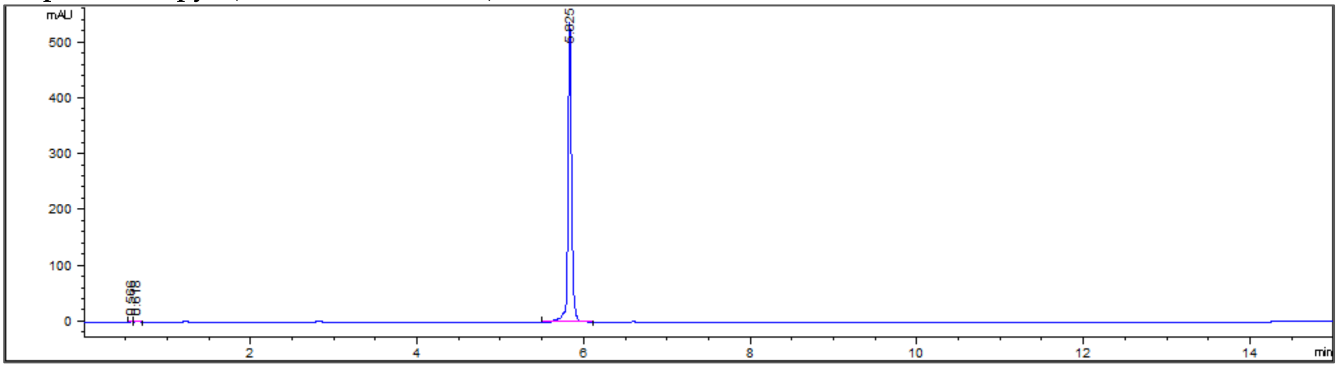

PHAM-Prop. (Rt = 6.60 min; 97.1%)

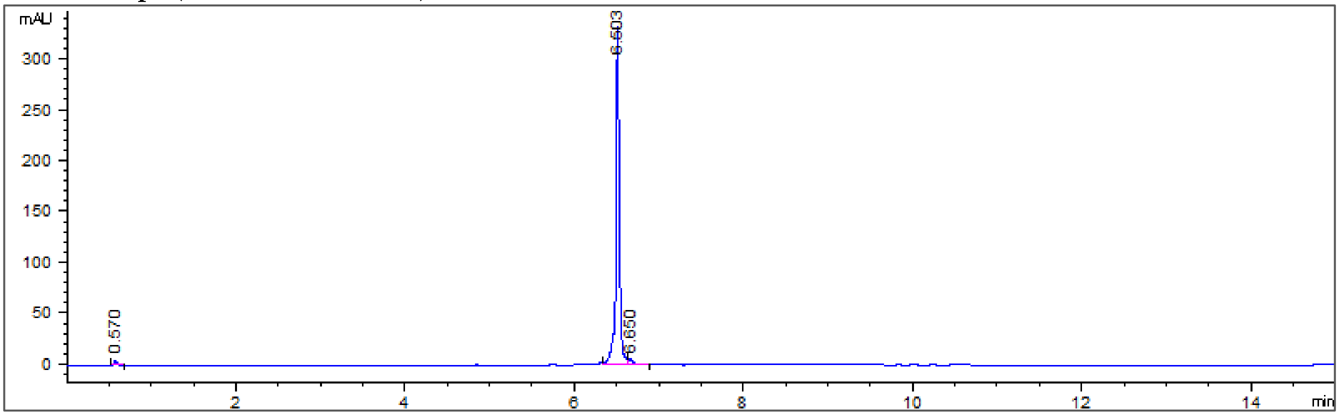

DIPAY. (Rt = 5.85 min; 99.95%)

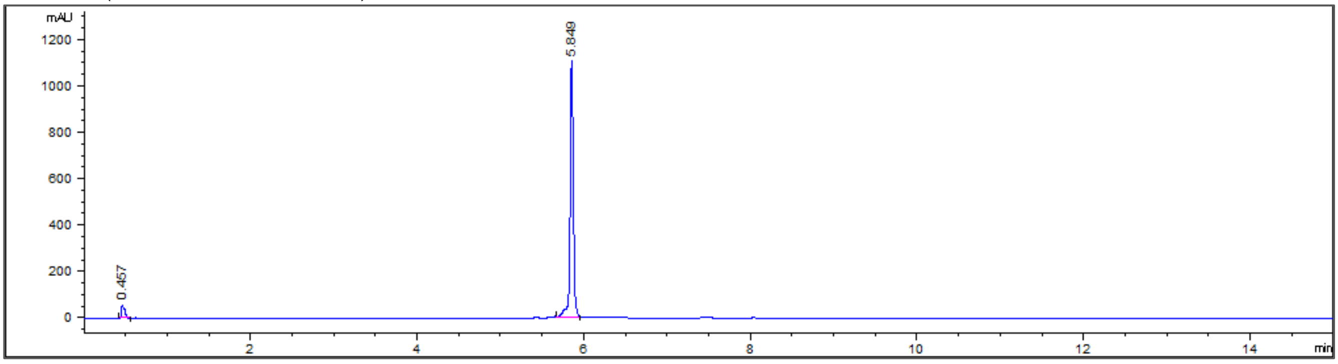

Compound 3. (Rt = 6.60 min; 91.3%)

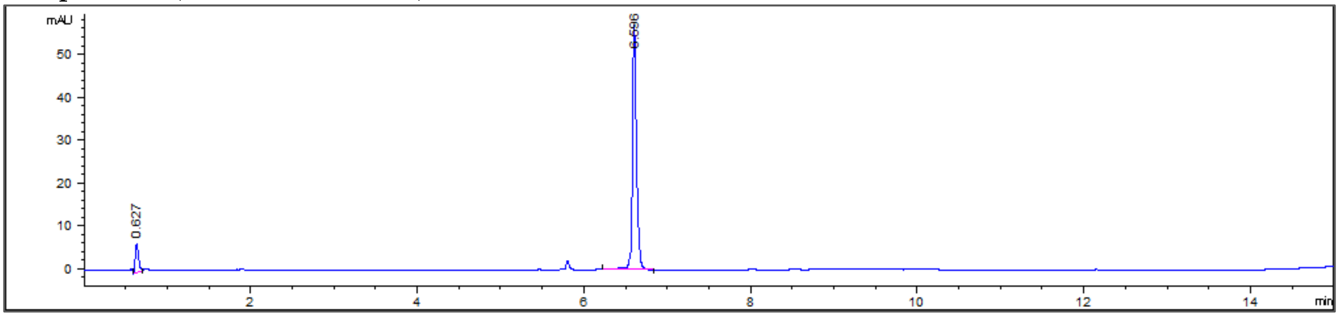

DIPAC. (Rt = 6.62 min; 100%)

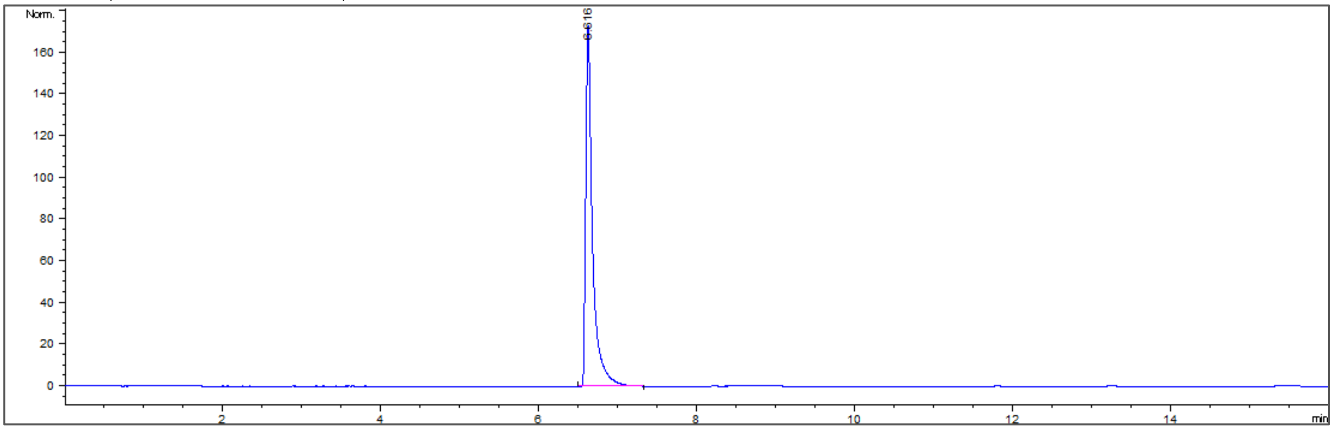

UHPLC-HRMS data. Positive mode acquisitions

PropNMe2Propyl. Found: 494.2749 m/z; precursor mass: 494.2762 m/z; mass error: -2.6 ppm.

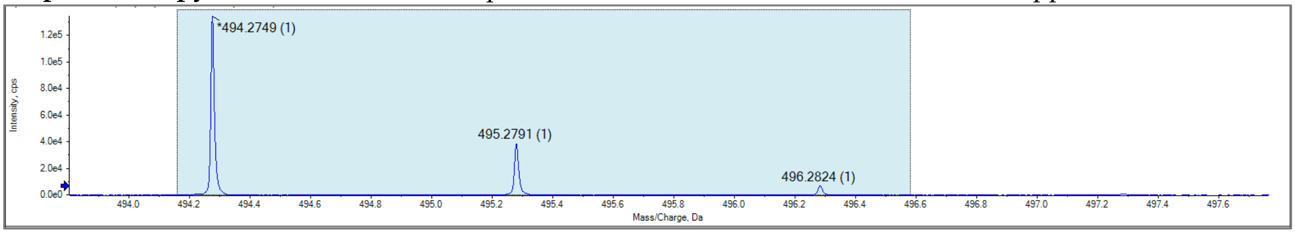

PHAM-Prop. Found: 625.3075 m/z; precursor mass: 625.3085 m/z; mass error: -1.7 ppm.

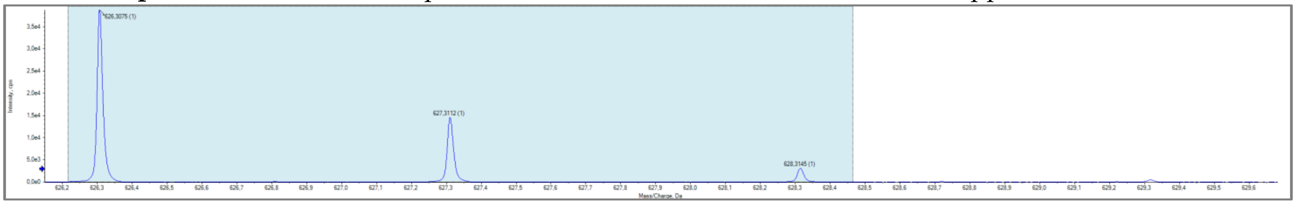

DIPAY. Found: 1131.5760 m/z; precursor mass: 1131.5774 m/z; mass error: -1.3 ppm.

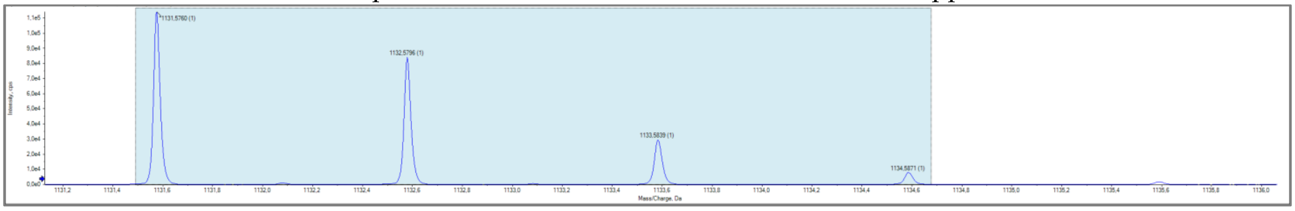

DIPAC. Found: 1156.5466 m/z; precursor mass: 1156.5475 m/z; mass error: -0.8 ppm.

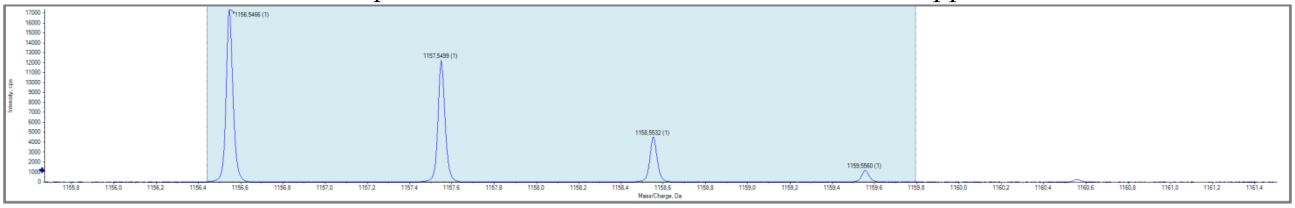

NMR data.

DIPAY. ^{1}H-NMR (300MHz, D_2O)

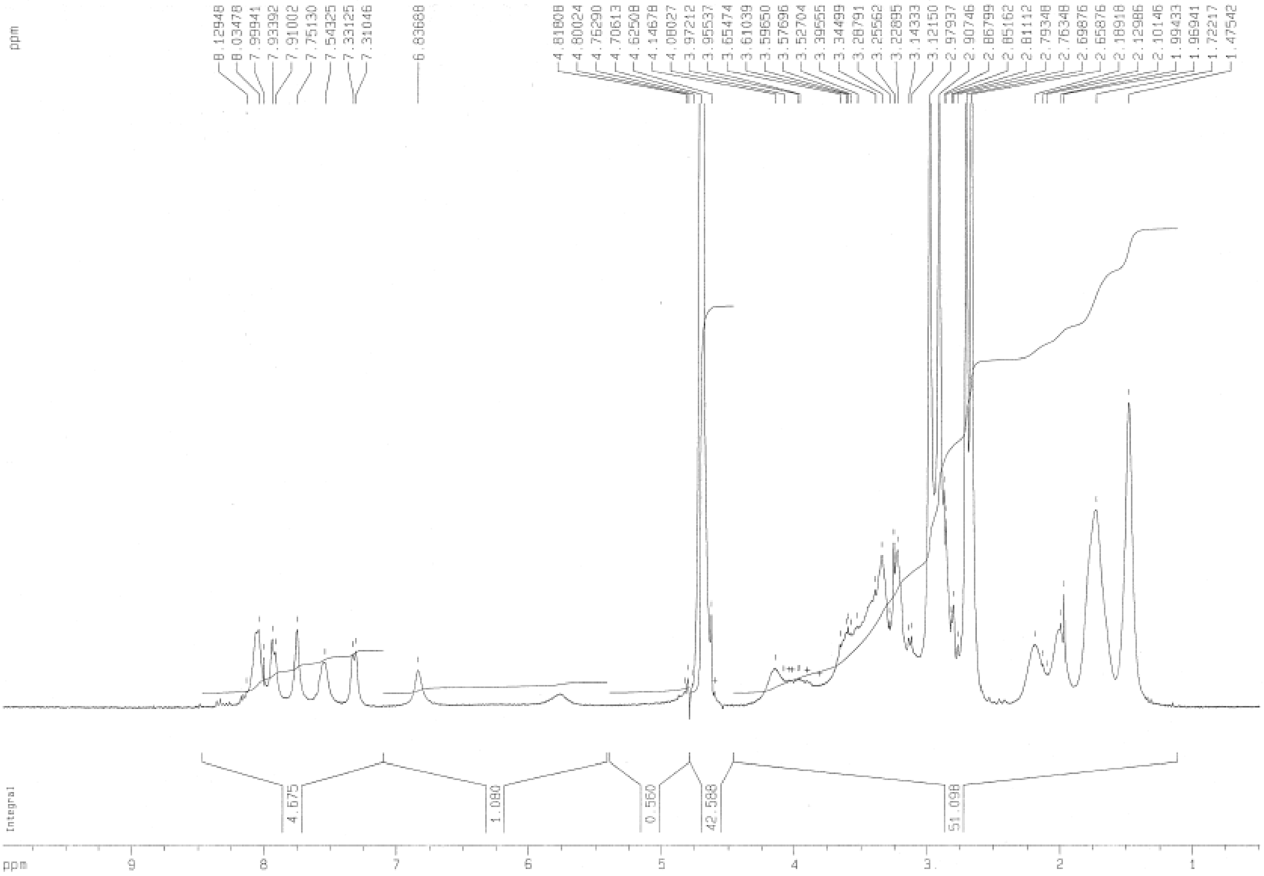

DIPAY. ^{13}C-NMR (75MHz, D_2O)

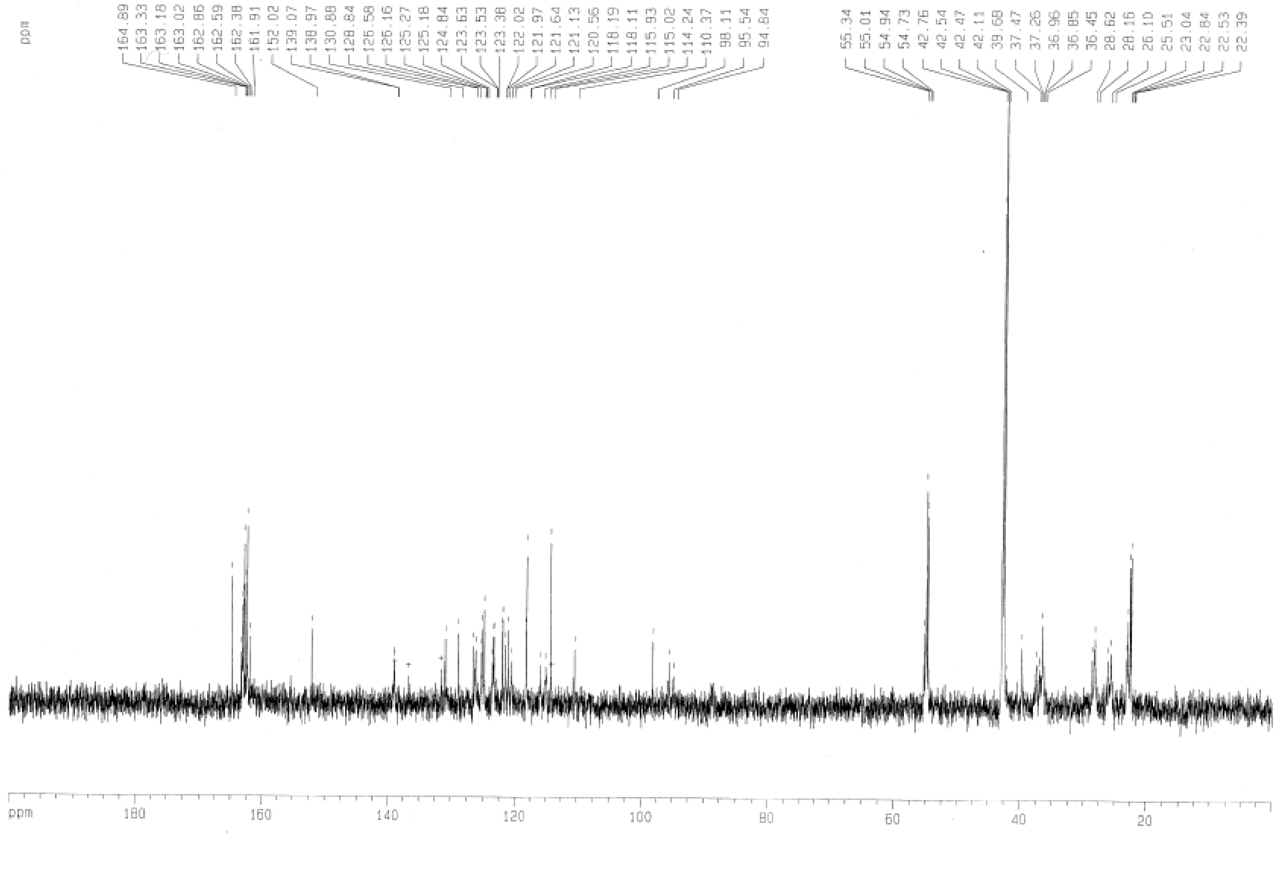

<u>HPHAMCON3.</u> ^{1}H-NMR (300MHz, D_2O)

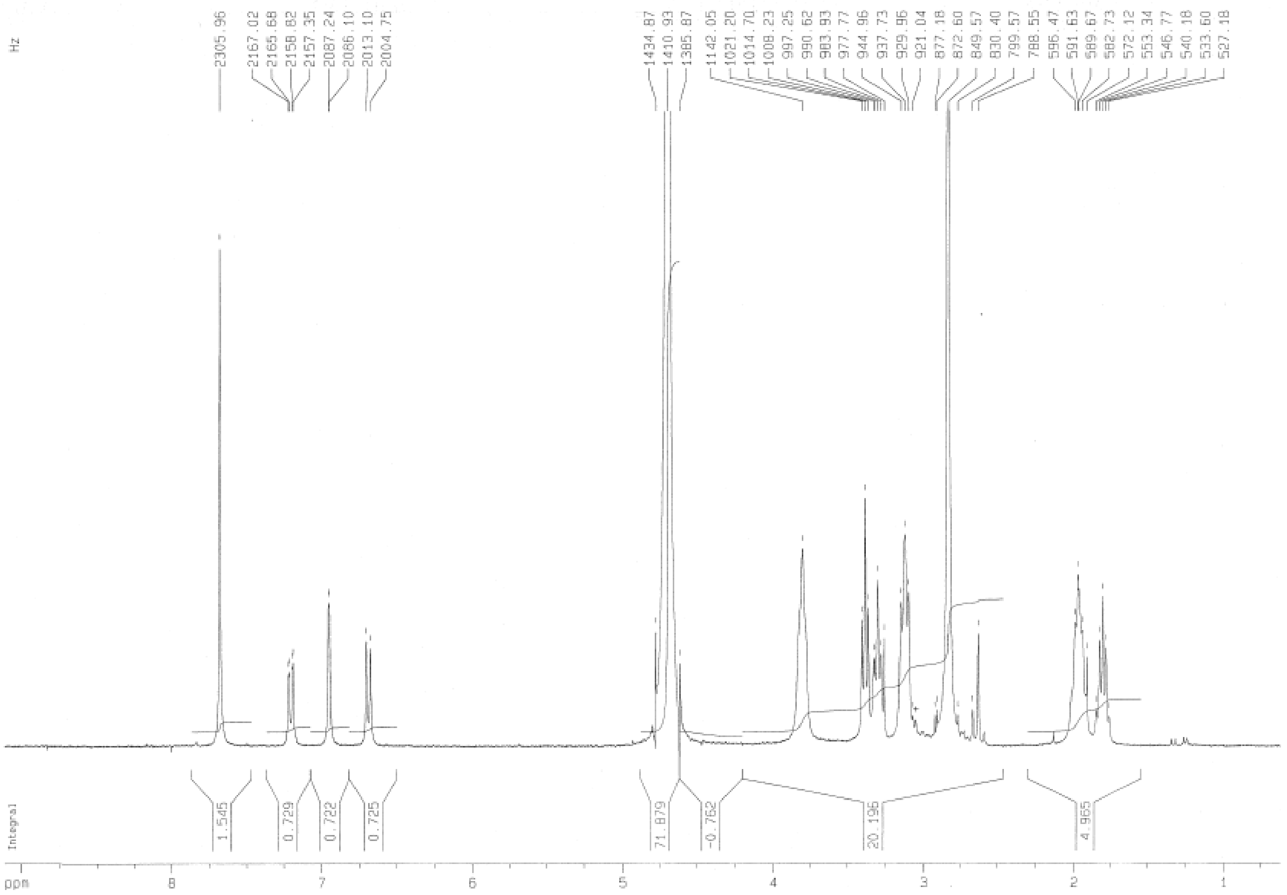

<u>HPHAMCON3.</u> ^{13}C-NMR (75MHz, D_2O)

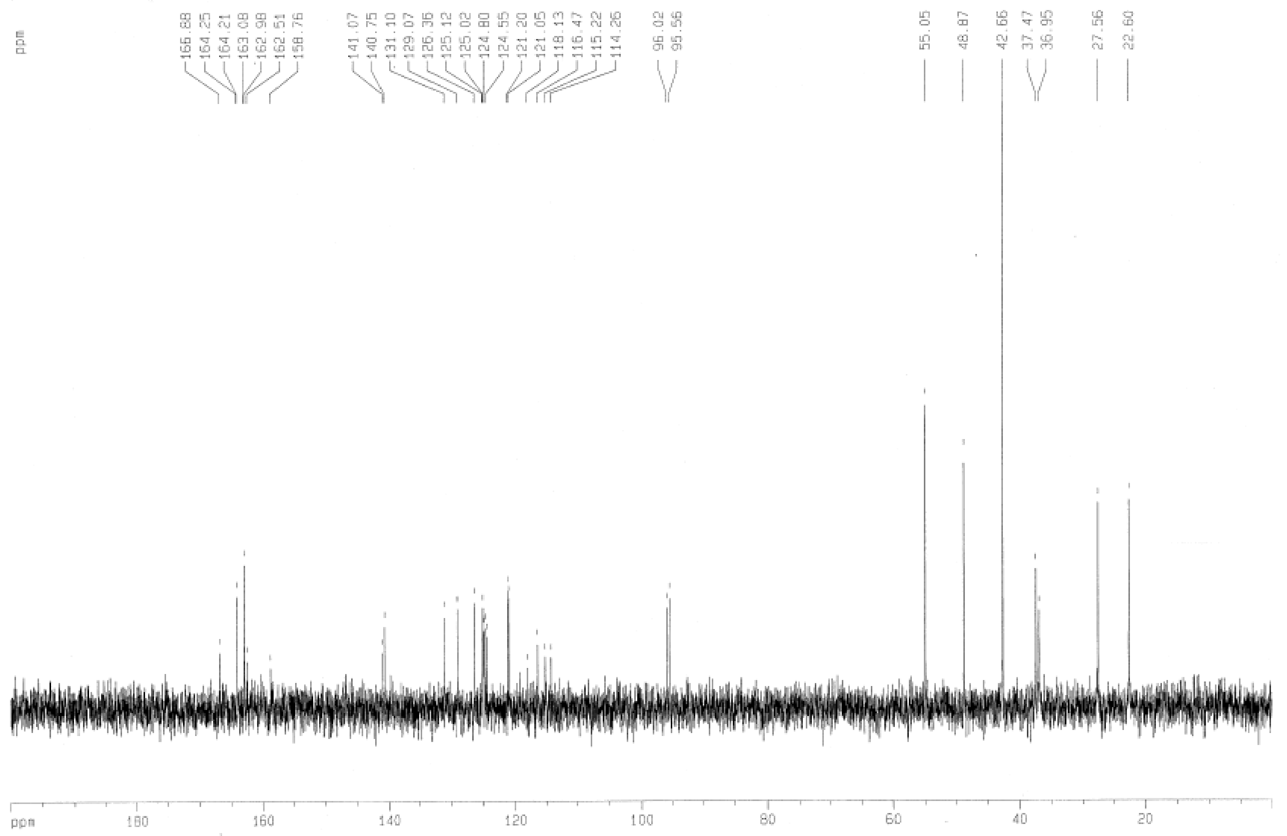

DIPAC. ^{1}H-NMR (300MHz, D_2O)

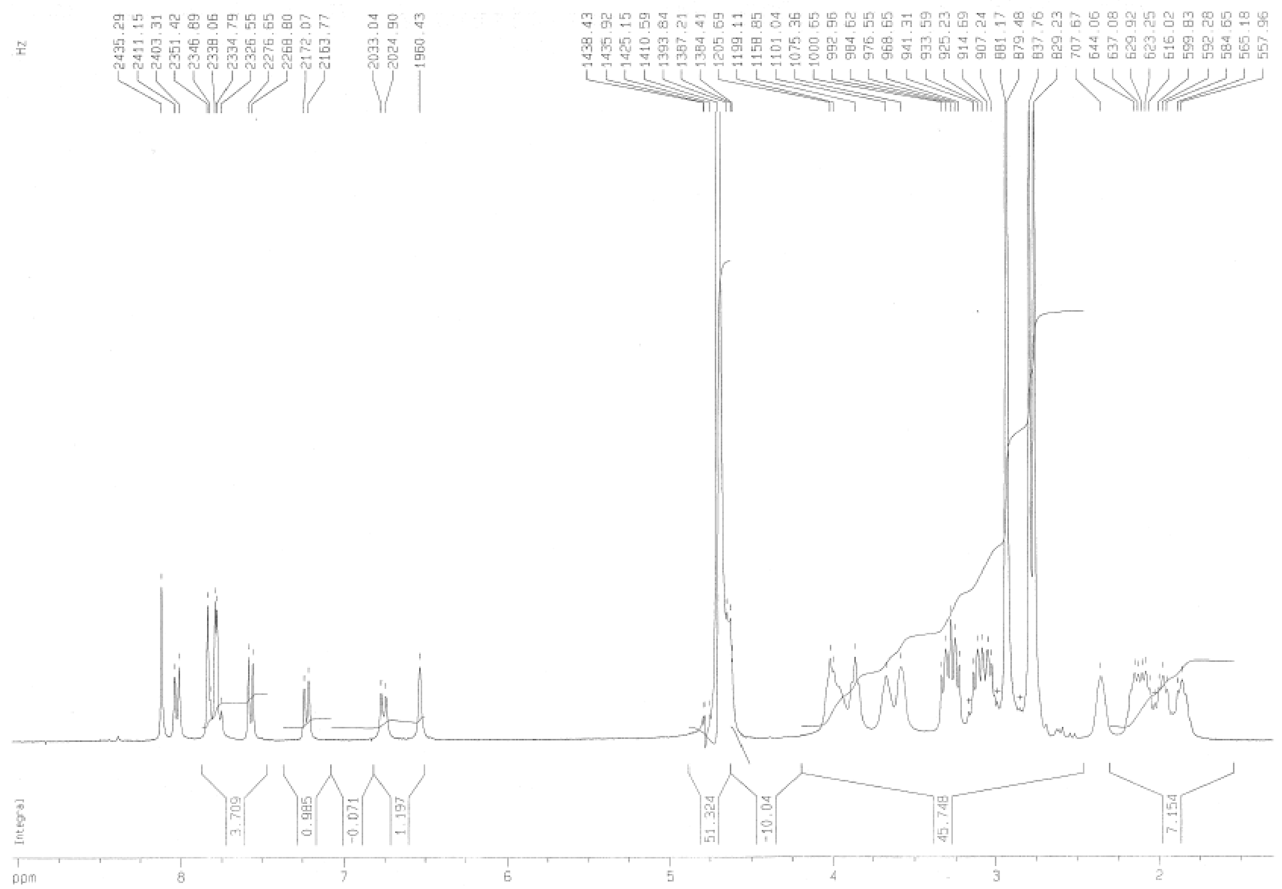

DIPAC. ^{13}C-NMR (75MHz, D_2O)

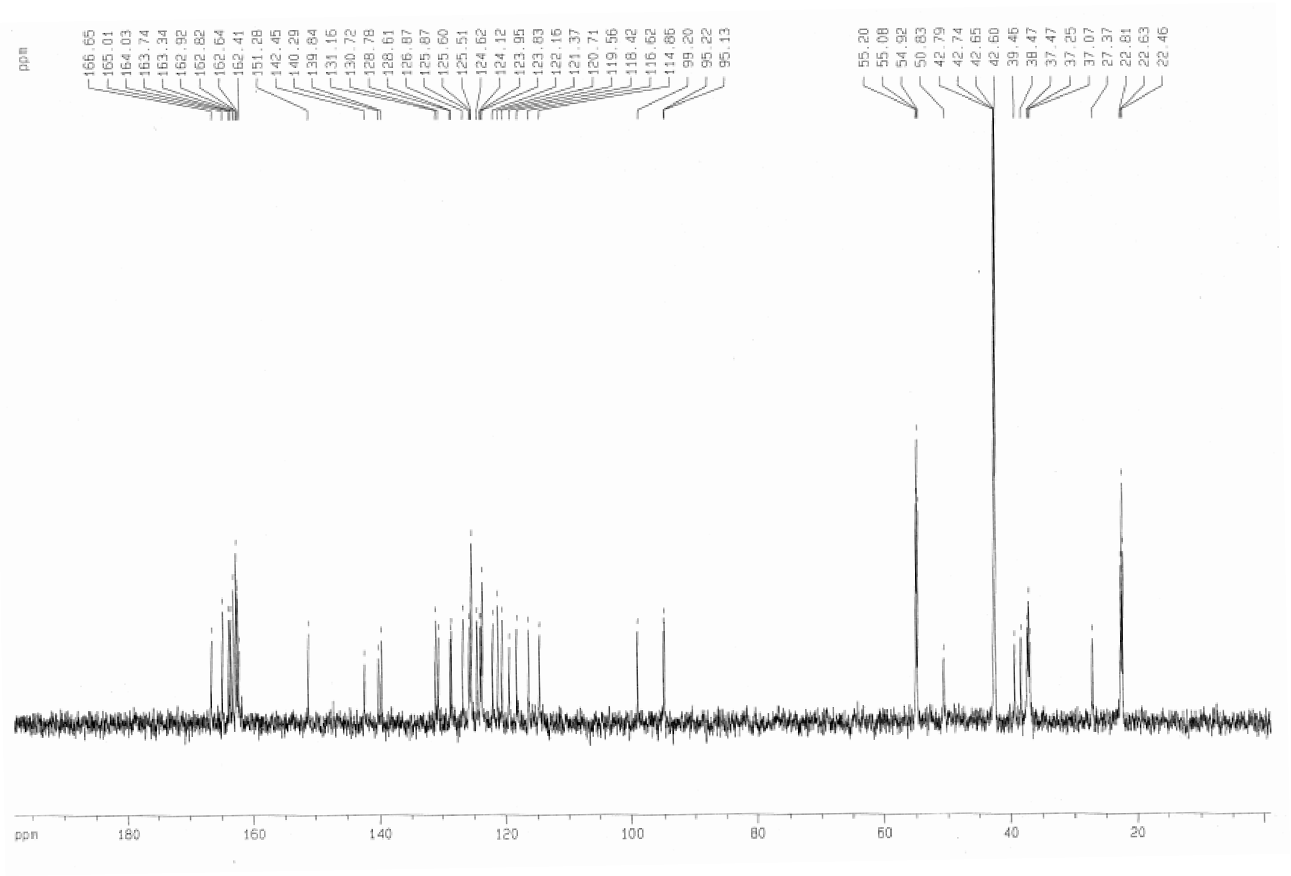

Supplement: Supplementary file 1 [file biomolecules-15-00311-s001.zip › biomolecules-3440638-supplementary.pdf]
